# pH-responsive activation of Tet-On inducible CAR-T cells enables spatially selective treatment of targeted solid tumors at reduced safety risk

**DOI:** 10.1093/nsr/nwaf306

**Published:** 2025-07-31

**Authors:** Yan Liu, Yu Hao, Jin Zhang, Mengmeng Zhang, Jiahui Chen, Zhengmiao Xia, Minming Chen, Xiang Lv, Xinxing Ma, Yehui Zhou, Jing Xu, Linqi Zhu, Wei Zhou, Liangzhu Feng

**Affiliations:** Jiangsu Key Laboratory for Molecular and Medical Biotechnology, Cancer Institute, Department of Biochemistry, College of Life Sciences, Nanjing Normal University, Nanjing 210023, China; Institute of Functional Nano & Soft Materials (FUNSOM), Jiangsu Key Laboratory for Carbon-Based Functional Materials & Devices, Soochow University, Suzhou 215123, China; Jiangsu Key Laboratory for Molecular and Medical Biotechnology, Cancer Institute, Department of Biochemistry, College of Life Sciences, Nanjing Normal University, Nanjing 210023, China; Jiangsu Key Laboratory for Molecular and Medical Biotechnology, Cancer Institute, Department of Biochemistry, College of Life Sciences, Nanjing Normal University, Nanjing 210023, China; Jiangsu Key Laboratory for Molecular and Medical Biotechnology, Cancer Institute, Department of Biochemistry, College of Life Sciences, Nanjing Normal University, Nanjing 210023, China; Jiangsu Key Laboratory for Molecular and Medical Biotechnology, Cancer Institute, Department of Biochemistry, College of Life Sciences, Nanjing Normal University, Nanjing 210023, China; Institute of Functional Nano & Soft Materials (FUNSOM), Jiangsu Key Laboratory for Carbon-Based Functional Materials & Devices, Soochow University, Suzhou 215123, China; Jiangsu Key Laboratory for Molecular and Medical Biotechnology, Cancer Institute, Department of Biochemistry, College of Life Sciences, Nanjing Normal University, Nanjing 210023, China; Department of Radiology, The First Affiliated Hospital of Soochow University, Suzhou 215006, China; Department of General Surgery, The First Affiliated Hospital of Soochow University, Suzhou 215000, China; Department of Clinical Laboratory, Zhongda Hospital Southeast University, Nanjing 210009, China; Department of Clinical Laboratory, The Third Affiliated Hospital of Soochow University, Changzhou 213003, China; Department of Clinical Laboratory, The Third Affiliated Hospital of Soochow University, Changzhou 213003, China; Institute of Functional Nano & Soft Materials (FUNSOM), Jiangsu Key Laboratory for Carbon-Based Functional Materials & Devices, Soochow University, Suzhou 215123, China; Department of Clinical Laboratory, The Third Affiliated Hospital of Soochow University, Changzhou 213003, China

**Keywords:** pH-responsive nanomedicine, doxycycline-inducible Tet-On system, spatially selective activation, tumor acidity neutralization, CAR-T-cell therapy

## Abstract

Chimeric antigen receptor T (CAR-T)-cell therapy is a promising resolution for solid tumors, but its corresponding clinical translation has been hindered by unsatisfactory therapeutic potency and severe cytokine release syndrome. Herein, tetracycline (Tet)-On inducible human epidermal growth factor receptor 1 (HER1)-targeted CAR-T (Tet-HER1-CAR-T) cells were engineered to enable spatially selective activation at tumor sites by doxycycline (Doxy), which is delivered by pH-responsive stealth liposomal calcium carbonate nanoparticles (Doxy@CaCO_3_-PEG). Compared with the intravenous administration of conventional HER1-CAR-T cells and Tet-HER1-CAR-T cells activated by free Doxy, concurrent intravenous administration of Tet-HER1-CAR-T cells and Doxy@CaCO_3_-PEG leads to the localized tumor activation of Tet-HER1-CAR-T cells and reduced systemic secretion of inflammatory cytokines. Together with its ability to protect Tet-HER1-CAR-T cells from tumor-acidity-induced dysfunction by neutralizing tumor acidity, Doxy@CaCO_3_-PEG injection synergized with Tet-HER1-CAR-T cells to effectively suppress the growth of HER1-overexpressing subcutaneous triple-negative breast cancer (TNBC) tumors, lung tumors and orthotopic lung tumors in mice. Furthermore, Doxy@CaCO_3_-PEG-activated Tet-HER1-CAR-T-cell therapy synergistically suppressed HER1 inhibitor-resistant TNBC tumors and immunosuppressive *Fusobacterium nucleatum (F.n.)* colonized HER1-overexpressing TNBC patient-derived tumor xenografts. This study highlights that the Doxy@CaCO_3_-PEG-induced pH-responsive activation of Tet-HER1-CAR-T cells is a highly spatially selective strategy for effectively eradicating targeted solid tumors with improved safety.

## Teaser

This study develops a pH-responsive CAR-T cell therapy activated only at tumor sites using CaCO_3_ based nanomedicine, effectively targeting aggressive solid tumors while minimizing harmful side effects.

## INTRODUCTION

Chimeric antigen receptor T (CAR-T)-cell therapy has emerged as a promising anticancer immunotherapy approach and eight CAR-T-cell therapies have already been approved for the clinical treatment of hematologic malignancies because of their impressive curative rates [1,2]. Considering the high selectivity of CAR-T cells in recognizing targeted cells, CAR-T-cell therapy is also considered a powerful and promising strategy for eradicating solid tumors—even intractable drug-resistant tumors [3,4]. However, the tumor-suppression efficacy of most available CAR-T cells toward solid tumors has been verified to be limited because the hostile tumor microenvironment (e.g. tumor acidity, immunosuppression and bacterial colonization) significantly impairs the tumor-infiltration and effector functions of CAR-T cells [5–7]. Recently, numerous strategies have been developed to potentiate CAR-T cells toward corresponding solid tumors by promoting their tumor infiltration and persistence via genetic engineering to express immunostimulatory cytokines (e.g. interleukin-7/10/15, C–C motif chemokine 19/21) and downregulate immunosuppressive molecules (e.g. programmed cell death protein 1, transforming growth factor-β) or modulating the tumor microenvironment with different functional biomaterials [8–14]. Therefore, exploring effective ways to enhance CAR-T-cell therapy by reprogramming hostile tumor microenvironments is highly practical.

In addition, engineered CARs commonly have high recognition affinity for corresponding targeted antigens to achieve selective and effective tumor elimination, but the low-level expression of these antigens in normal tissues in turn causes unwanted cytokine release syndrome [15,16]. Learning from the principles of designing selectively activatable prodrugs, developing strategies that enable the tumor-selective activation of CAR-T cells is promising for eliminating solid tumors with improved therapeutic effectiveness and safety [17]. Among them, the tetracycline (Tet)-On system, which is an inducible gene expression system controlled by the reverse Tet transactivator (rtTA) comprising a doxycycline (Doxy)-binding Tet-repressor mutant protein and a C-terminal activator domain, has recently been explored to engineer Doxy-activatable CAR-T cells for treating blood tumors [18,19]. However, owing to the limited tumor accumulation efficacy of Doxy in solid tumors, there are no reports on the engineering of Tet-On CAR-T cells for the treatment of solid tumors. Therefore, developing suitable approaches that enable tumor-targeted delivery of Doxy is a promising approach to selectively activate engineered Tet-On CAR-T cells for the specific treatment of solid tumors without causing systemic cytokine release syndrome.

Calcium carbonate (CaCO_3_) nanoparticles with excellent biocompatibility, versatile ion/molecule-loading capacity and proton-scavenging capacity have been widely explored to potentiate diverse cancer treatments against solid tumors by enabling pH-dependent drug delivery and/or tumor acidity neutralization [20,21]. In this study, long-circulating liposomal CaCO_3_ nanoparticles prepared according to our previously developed method were employed to enable the simultaneous tumor-targeted delivery of Doxy and neutralization of tumor acidity. The obtained Doxy@CaCO_3_-PEG-activated engineered Tet-human epidermal growth factor receptor 1 (HER1)-CAR-T cells with comparable efficacy and protected them from acidity incubation-induced dysfunction *in vitro*. Upon intravenous injection, Doxy@CaCO_3_-PEG enabled the spatially selective activation of tumor-infiltrating Tet-HER1-CAR-T cells without causing any obvious secretion of pro-inflammatory cytokines (e.g. interleukin 2 (IL-2), interferon-γ (IFN-γ) and tumor necrosis factor-α (TNF-α)) into the blood compared with that in mice treated with free Doxy-activated Tet-HER1-CAR-T cells and conventional HER1-CAR-T cells. Consequently, treatment with sequential injections of Doxy@CaCO_3_-PEG and Tet-HER1-CAR-T cells synergistically inhibited the growth of HER1-overexpressing subcutaneous triple-negative breast cancer (TNBC), lung cancer, erlotinib-resistant TNBC and orthotopic lung cancer. Inspired by the intrinsic antibacterial function of Doxy, Doxy@CaCO_3_-PEG could also potentiate the therapeutic efficacy of Tet-HER1-CAR-T cells against immunosuppressive *Fusobacterium nucleatum (F.n.)*-colonized HER1-overexpressing TNBC patient-derived tumor xenografts (PDXs). This study highlights that Doxy@CaCO_3_-PEG is a promising candidate for enabling the concurrent tumor-localized activation of engineered CAR-T cells, tumor acidity neutralization and removal of immunosuppressive *F.n.* for improved treatment of targeted solid tumors with reduced safety risk.

## RESULTS

### Preparation and characterization of Doxy@CaCO_3_-PEG

**Figure 1.**
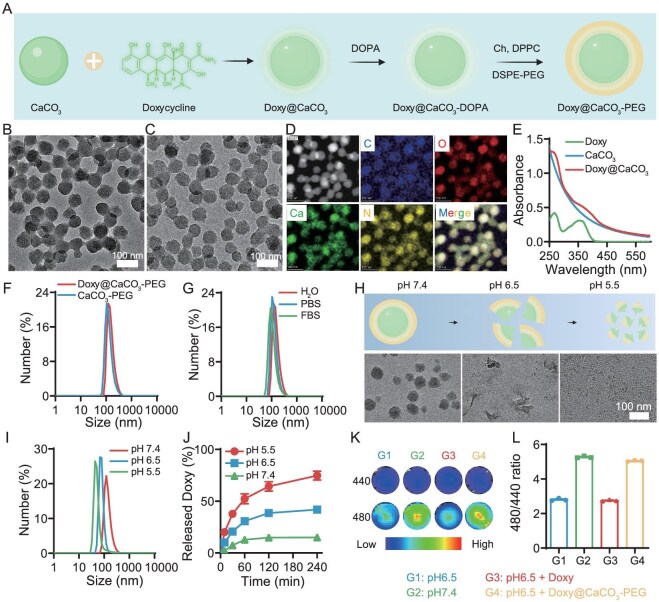
Preparation and characterization of Doxy@CaCO_3_-PEG. (A) Scheme showing the synthesis and surface modification process of Doxy@CaCO_3_-PEG. (B, C) Representative TEM images of (B) CaCO_3_ nanoparticles and (C) Doxy@CaCO_3_. (D) STEM image of the as-prepared Doxy@CaCO_3_. (E) UV‒vis absorption curves of Doxy, CaCO_3_ and Doxy@CaCO_3_. (F) Hydrodynamic size distributions of CaCO_3_-PEG and Doxy@CaCO_3_-PEG. (G) Hydrodynamic diameter distribution profiles of Doxy@CaCO_3_-PEG incubated in various physiological solutions as indicated for 24 h. (H) TEM images of Doxy@CaCO_3_-PEG incubated in buffer solutions at different pH values (5.5, 6.5 and 7.4) for 4 h. (I) Hydrodynamic size distribution profiles of Doxy@CaCO_3_-PEG incubated in buffer solutions at different pH values (5.5, 6.5 and 7.4) for 4 h. (J) Time-dependent release profiles of Doxy from Doxy@CaCO_3_-PEG incubated in buffer solutions at different pH values as indicated. (K, L) pH-responsive ratiometric BCECF fluorescence images with excitation wavelengths of 440 and 480 nm for different solutions as indicated (K), as well as semi-quantitative analysis of their corresponding fluorescence intensity ratios (L).

To enable tumor-targeted delivery of Doxy for the tumor-localized activation of Tet-HER1-CAR-T cells, amorphous CaCO_3_ nanoparticles that are efficient at loading small molecules were synthesized, loaded with Doxy and coated with lipid bilayers to render their superior tumor-homing capacity according to our previously developed methods [22] (Fig. 1A). The as-prepared amorphous CaCO_3_ nanoparticles showed a uniform spherical morphology via transmission electron microscopy (TEM) (Fig. 1B). Attributed to the strong coordination interaction between Doxy and Ca^2+^, CaCO_3_ nanoparticles showed a high loading efficiency for Doxy molecules (∼80%) and the loading of Doxy had a minimal influence on their morphology (Fig. 1C and Fig. S1). As characterized by elemental mapping via high-angle annular dark field scanning TEM (HAADF-STEM), the obtained Doxy@CaCO_3_ nanoparticles clearly presented C, N, O and Ca signals (Fig. 1D). The successful loading of Doxy was further confirmed by the considerably increased absorbance in the range of 250–450 nm, which overlapped with the typical absorption of Doxy in the ultraviolet–visible–near-infrared (UV–vis–NIR) spectrum of Doxy@CaCO_3_ (Fig. 1E). The results of dynamic light scattering (DLS) measurements indicated that Doxy@CaCO_3_-PEG exhibited monodispersed size distribution profiles comparable to those of CaCO_3_-PEG and, after being incubated in fetal bovine serum (FBS) solution, phosphate-buffered saline (PBS) or H_2_O was added for ≤24 h (Fig. 1F and G), confirming its excellent physiological stability.

Owing to the intrinsic pH-responsive dissociation capacity of CaCO_3_, Doxy@CaCO_3_-PEG exhibited rapid pH-responsive destruction, as indicated by the disappearance of the typical CaCO_3_ nanoparticle morphology under TEM imaging and size shrinkage via DLS measurements after incubation at pH 5.5 and 6.5 for 4 h (Fig. 1H and I). Doxy@CaCO_3_-PEG exhibited pH-responsive release behavior for Doxy and 75%, 42% and 15% Doxy were released after incubation at pH 5.5, 6.5 and 7.4 for 4 h, respectively (Fig. 1J). Via ratiometric fluorescence imaging with the pH-sensitive fluorescent dye 2,7-biscarboxyethyl-5(6)-carboxyfluorescein (BCECF), Doxy@CaCO_3_-PEG was shown to efficiently neutralize the acidic solution (2 mM lactic acid) (Fig. 1K and L). Collectively, these results indicate that Doxy@CaCO_3_-PEG is able to enable pH-responsive Doxy delivery and the neutralization of tumor acidity.

### Doxy@CaCO_3_-PEG activates engineered Tet-HER1-CAR-T cells and protects them from acidity-induced dysfunction

**Figure 2.**
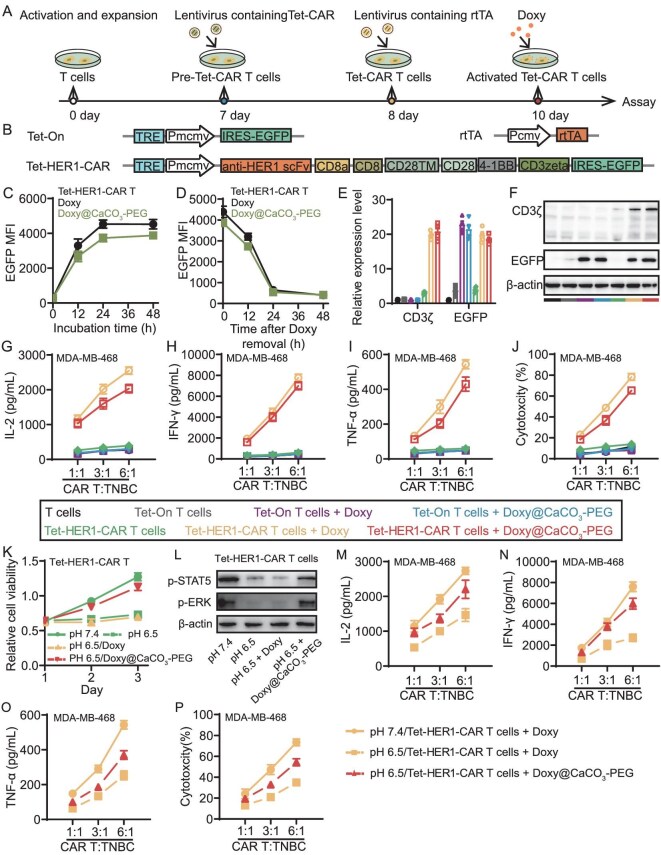
Doxy@CaCO_3_-PEG activates Tet-HER1-CAR-T cells and protects them from acidity-induced exhaustion. (A) Scheme showing the experimental schedule for the generation of Tet-HER1-CAR-T cells and their activation by Doxy incubation. (B) Structural diagram of the Tet-On, Tet-HER1-CAR and rtTA used for generating the Tet-HER1-CAR-T cells. Note that the Tet-HER1-CAR comprises: antigen recognition domain (anti-HER1-scFv), hinge (CD8α/CD8), transmembrane region (CD28TM), co-stimulatory domains (CD28/4–1BB) and signaling domain (CD3ζ). (C, D) Flow cytometric analysis of time-dependent EGFP expression in Tet-HER1-CAR-T cells (C) treated with Doxy, Doxy@CaCO_3_-PEG or (D) treated with Doxy, Doxy@CaCO_3_-PEG for 24 h, then Doxy removed from the medium at the indicated times. ([Doxy] = 1 μg/mL). (E) RT‒PCR and (F) Western-blot analyses of CD3ζ and EGFP expression in Tet-On T cells and Tet-HER1-CAR-T cells after different treatments as indicated. (G) IL-2 , (H) IFN-γ and (I) TNF-α secretion abilities and cytolysis ability (J) of Tet-HER1-CAR-T cells or other T cells co-incubated with MDA-MB-468 cells at various feed ratios as indicated and Doxy or Doxy@CaCO_3_-PEG ([Doxy] = 1 μg/mL) for 24 h. (K) Relative viability of Tet-HER1-CAR-T cells after different treatments as indicated. (L) Western-blot analyses of p-STAT5 and p-ERK expression in Tet-HER1-CAR-T cells after different treatments as indicated for 24 h. (M–P) Cytokine secretion (M–O) and cytolytic ability of Tet-HER1-CAR-T cells co-cultured with MDA-MB-468 cells at different ratios as indicated and with Doxy or Doxy@CaCO_3_-PEG ([Doxy] = 1 μg/mL) for 24 h.

To generate Doxy-inducible Tet-HER1-CAR-T cells (Fig. 2A), the third-generation HER1-CAR-expressing sequence containing the anti-HER1-specific single‐chain variable fragment, CD8a, CD8, CD28TM, CD28, 4–1BB and CD3ζ genes was first inserted into the Tet-On system expressing lentiviral vectors between the restriction enzyme cutting sites of *NdeI* and *PspXI* (Fig. 2B). The successful construction of the Tet-HER1-CAR vector was verified by using standard *NdeI/PspXI* restriction digestion and gel electrophoresis (Fig. S2). The naive T cells isolated from healthy human donors were subsequently simultaneously transduced with lentiviral vectors carrying Tet-HER1-CAR and rtTA sequences to generate Tet-HER1-CAR-T cells. Moreover, the isolated human T cells were transduced with lentiviral vectors carrying an empty Tet-On system and an rtTA sequence to generate Doxy-activatable nonspecific Tet-On T cells. The successful engineering of Tet-On T cells and Tet-HER1-CAR-T cells was confirmed by recording the expression of enhanced green fluorescence protein (EGFP) in the Tet-On system and of CD3ζ in the presence of Doxy stimulation via flow cytometry, real-time polymerase chain reaction (RT‒PCR) and Western blot, respectively.

The incubation with Doxy@CaCO_3_-PEG or Doxy led to efficient EGFP expression by both Tet-On T cells and Tet-HER1-CAR-T cells in a time- and Doxy-concentration-dependent manner (Fig. S3A and B). The incubation with Doxy@CaCO_3_-PEG or Doxy at 1 μg/mL (in terms of Doxy) for 24 h was selected for subsequent *in vitro* cell experiments to ensure the maximal activation levels of the engineered T cells (>90%) (Fig. 2C and Fig. S3C and D). Furthermore, the activated T cells were deactivated upon incubation in fresh cell culture medium for ∼24 h, as indicated by markedly reduced EGFP expression via flow cytometry (Fig. 2D). The RT‒PCR and Western-blot results indicated that the Tet-HER1-CAR-T cells exhibited Doxy-dependent expression of EGFP and CD3ζ, whereas the Tet-On T cells only exhibited Doxy-dependent expression of EGFP (Fig. 2E and F). In addition, via cell viability measurements, we found that both Doxy and Doxy@CaCO_3_-PEG nanoparticles did not impair the viability of MDA-MB-468, NCI-H23, T cells, Tet-On T cells or Tet-HER1-CAR-T cells (Fig. S4). Doxy@CaCO_3_-PEG-activated Tet-On T cells or Tet-HER1-CAR-T cells exhibited similar proliferation rates to their counterpart T cells and naive T cells (Fig. S5). These results collectively indicate that the engineered Tet-HER1-CAR-T cells could be specifically activated by Doxy@CaCO_3_-PEG.

We then investigated the capacity of the Tet-HER1-CAR-T cells in the presence of Doxy@CaCO_3_-PEG and HER1-overexpressing tumor cells to secrete pro-inflammatory cytokines and kill targeted cells. The enzyme-linked immunosorbent assay (ELISA) results revealed that the Tet-HER1-CAR-T cells activated by Doxy and Doxy@CaCO_3_-PEG in the presence of HER1-overexpressing TNBC (MDA-MB-468) and lung cancer (NCI-H23) cells effectively secreted IL-2, IFN-γ and TNF-α at comparable levels (Fig. 2G–I and Fig. S6A–C). In sharp contrast, Tet-HER1-CAR-T cells incubated with only HER1-expressing cancer cells and Tet-On-T cells incubated with HER1-overexpressing cancer cells in the presence of Doxy or Doxy@CaCO_3_-PEG presented negligible secretion of these cytokines. In addition, cytolytic assays revealed that only the Tet-HER1-CAR-T cells activated by Doxy and Doxy@CaCO_3_-PEG effectively lysed the co-cultured MDA-MB-468 and NCI-H23 cells (Fig. 2J and Fig. S6D). Furthermore, upon activation by Doxy@CaCO_3_-PEG ([Doxy] = 1 μg/mL, 24 h), the engineered Tet-HER1-CAR-T cells presented similar cytokine secretion and cytolytic capacity to those of normal HER1-CAR-T cells (Fig. S7). The results demonstrated that Doxy@CaCO_3_-PEG could activate Tet-HER1-CAR-T cells to specifically lyse HER1-overexpressing tumor cells *in vitro*.

Previous studies have shown that tumor acidity—a general microenvironmental feature of solid tumors—is a main cause of the accumulation of anergic tumor-infiltrating T cells with impaired cell viability, cytokine secretion and cytolytic activity by suppressing the activation of the signal transducer and activator of transcription 5A–extracellular regulated protein kinase (STAT5–ERK) axis [23]. A cell proliferation assay revealed that acidic incubation (lactic acid, pH 6.5, 48 h) significantly suppressed the proliferation rates of both Tet-On T cells and Tet-HER1-CAR-T cells compared with their counterparts under normal incubation conditions (pH 7.4). However, the acidic incubation exhibited negligible disturbance on the viability of the MDA-MB-468 and NCI-H23 cells (Fig. S8). Owing to the proton-scavenging capacity of CaCO_3_, Doxy@CaCO_3_-PEG incubation effectively reversed the acidic incubation-induced suppression of the proliferation of the engineered T cells (Fig. 2K). As expected, free Doxy treatment had no ability to reverse the suppressive effect of acidic incubation on the proliferation of these engineered T cells. Moreover, Western blot revealed that Doxy@CaCO_3_-PEG incubation reversed the acidic incubation-induced decrease in the phosphorylation of STAT5 and ERK, which was correlated with the survival of Tet-On T cells and Tet-HER1-CAR-T cells (Fig. 2L).

After that, acidic incubation markedly suppressed the secretion of IL-2, IFN-γ and TNF-α by Tet-HER1-CAR-T cells co-cultured with free Doxy and MDA-MB-468 cells at pH 6.5 compared with those of Tet-HER1-CAR-T cells co-cultured with free Doxy and MDA-MB-468 cells at pH 7.4 (Fig. 2M-O). In contrast, Tet-HER1-CAR-T cells incubated with Doxy@CaCO_3_-PEG and MDA-MB-468 cells at pH 6.5 exhibited significantly increased secretion of the corresponding cytokines, which can be attributed to Doxy@CaCO_3_-PEG-mediated acidity neutralization. Consistently, Tet-HER1-CAR-T cells activated by Doxy@CaCO_3_-PEG also exhibited much greater lytic capacity to target MDA-MB-468 cells than did those activated by free Doxy at pH 6.5 (Fig. 2P). These results demonstrate that the proton-scavenging capacity of Doxy@CaCO_3_-PEG endows it with a superior ability to protect T cells from tumor acidity-induced anergy, thereby enabling the effective activation of Tet-HER1-CAR-T cells at low pH.

### Doxy@CaCO_3_-PEG enables spatially selective activation of Tet-HER1-CAR-T cells

**Figure 3.**
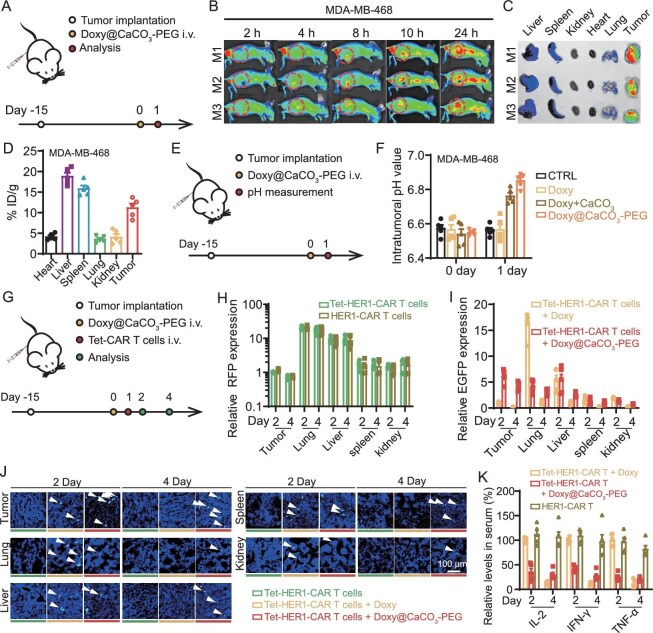
Doxy@CaCO_3_-PEG can neutralize tumor acidity and selectively activate Tet-HER1-CAR-T cells without inducing systemic cytokine secretion. (A) Scheme showing the experimental schedule for evaluating the *in vivo* pharmacokinetic profiles of Doxy@CaCO_3_-PEG. (B) *In vivo* time-lapse fluorescence images of MDA-MB-468-tumor-bearing mice intravenously injected with DiD-Doxy@CaCO_3_-PEG. (C) *Ex vivo* fluorescence images of MDA-MB-468 tumors and the main organs of DiD-Doxy@CaCO_3_-PEG-treated mice collected at 24 h post injection. (D) Semi-quantitative analysis of the biodistribution profiles of DiD-Doxy@CaCO_3_-PEG in MDA-MB-468-tumor-bearing mice at 24 h determined by recording the DiD fluorescence intensity of these homogenized organs and tumors. (E) Scheme showing the experimental schedule for evaluating the tumor acidity neutralization capacity of Doxy@CaCO_3_-PEG. (F) Intratumoral pH values of MDA-MB-468-tumor-bearing mice at 0 and 1 day after different treatments as indicated. (G) Scheme showing the experimental schedule for evaluating the *in vivo* biodistribution, activation and safety profiles of the Tet-HER1-CAR-T cells. (H) *In vivo* biodistribution profiles of HER1-CAR-T cells and Tet-HER1-CAR-T cells in MDA-MB-468-tumor-bearing mice determined by recording their RFP tag via RT‒PCR. (I) RT‒PCR and (J) immunofluorescence staining analysis of EGFP expression in the tumors and main organs of MDA-MB-468-tumor-bearing mice at 2 and 4 days after different treatments as indicated. (K) Relative serum cytokine levels in MDA-MB-468-tumor-bearing mice after different treatments as indicated.

The *in vivo* pharmacokinetic profiles of Doxy@CaCO_3_-PEG via intravenous injection were evaluated in MDA-MB-468 and NCI-H23 tumor-bearing nude mice (Fig. 3A). Via IVIS Lumina III *in vivo* fluorescence imaging, Doxy@CaCO_3_-PEG labeled with the lipophilic fluorescence probe 1,1′-dioctadecyl-3,3,3′,3′-tetramethylindodicarbocyanine,4-chlorobenzenesulfonate (DiD) gradually homed to tumor sites, as indicated by the gradually increased DiD fluorescence in the tumor region (Fig. 3B). Via *ex vivo* imaging, the tumors collected from the mice at 24 h after intravenous injection of DiD-labeled Doxy@CaCO_3_-PEG showed much stronger DiD fluorescence than did the main organs (Fig. 3C and Fig. S9). The homogenous distribution of Doxy@CaCO_3_-PEG inside tumors was further evaluated by recording the DiD fluorescence of tumor slices via confocal microscopy (Fig. S10). Semi-quantitative analysis revealed that the amount of DiD-labeled Doxy@CaCO_3_-PEG that accumulated in the MDA-MB-468 tumors 24 h after injection reached ∼10.67 ± 2.33% of the injected dose per gram of tissue (ID g^−1^) by recording the DiD fluorescence intensity of the homogenized organs (Fig. 3D and Fig. S11). Meanwhile, its accumulation amounts in liver and spleen were quantified to be ∼18.82 ± 2.33 and ∼15.82 ± 2.68% ID g^−1^, respectively. The distinct Doxy@CaCO_3_-PEG accumulation amounts of in livers and spleens shown in Fig. 3C and D should be ascribed to the fact that the dark color of these two organs would quench the fluorescence of the DiD during the *ex vivo* imaging according to a previous study [21]. In addition, the first half-life (*t*_1/2α_) and second half-life (*t*_1/2β_) of the DiD-labeled Doxy@CaCO_3_-PEG in MDA-MB-468-tumor-bearing mice were measured to be 1.43 ± 0.14 and 14.29 ± 2.06 h, respectively (Fig. S12A). Moreover, DiD-labeled Doxy@CaCO_3_-PEG also exhibited similar tumor accumulation efficacy and blood circulation times in NCI-N23-tumor-bearing mice (Fig. S12B). These results suggest that Doxy@CaCO_3_-PEG has a good stealth-like blood circulation profile and high passive tumor accumulation efficacy.

After that, we evaluated the ability of Doxy@CaCO_3_-PEG to neutralize tumor acidity by directly measuring the pH values inside the tumors via a commercial pH microelectrode. The intratumoral pH of the MDA-MB-468-tumor model mice intravenously injected with Doxy@CaCO_3_-PEG or a mixture of free Doxy and CaCO_3_-PEG increased from the original value of ∼6.5 to ∼6.9 at 24 h after the corresponding injection (Fig. 3E and F). Intravenous injections of PBS or free Doxy minimally disturbed the intratumoral pH of the treated mice. Together with the capacity of Doxy@CaCO_3_-PEG to neutralize the tumor acidity of NCI-N23 tumors (Fig. S13), Doxy@CaCO_3_-PEG potently neutralizes tumor acidity by utilizing the proton-scavenging capacity of CaCO_3_. Considering that tumor cells continuously secrete proton, lactate and carbon dioxide into the extracellular tumor microenvironments [24], tumor-accumulated Doxy@CaCO_3_-PEG is hypothesized to undergo gradual degradation alongside engulfment by cancer cells (Fig. S14), thereby enabling sustained Doxy release for the effective localized activation of Tet-HER1-CAR-T cells within tumors.

Next, we investigated the capacity of Doxy@CaCO_3_-PEG to promote the selective activation and persistence of Tet-HER1-CAR-T cells in tumors (Fig. 3G). By recording the expression of red fluorescence protein (RFP), which is expressed by both the Tet-HER1-CAR-T cells and conventional HER1-CAR-T cells via RT‒PCR, both CAR-T cells after intravenous injection exhibited similar tumor-infiltration efficacy but were less effective than those in the lungs and liver (Fig. 3H). By recording the expression of EGFP, an indicator of the activation of Tet-HER1-CAR-T cells via RT‒PCR, the tumors collected from the MDA-MB-468-tumor-bearing mice intravenously injected with the Tet-HER1-CAR-T cells Doxy@CaCO_3_-PEG presented higher EGFP expression levels than those from the mice treated with the Tet-HER1-CAR-T cells and Doxy at Days 2 and 4 (Fig. 3I). Moreover, unlike Doxy-activated Tet-HER1-CAR-T cells, which led to a high expression of EGFP in the lungs on Day 2, the mice treated with Tet-HER1-CAR-T cells and Doxy@CaCO_3_-PEG injections presented much lower EGFP expression in their lungs. The selective and sustained activation of Tet-HER1-CAR-T cells in tumors by Doxy@CaCO_3_-PEG was further verified via confocal microscopy (Fig. 3J). These results collectively suggest that Doxy@CaCO_3_-PEG can promote the selective and sustained activation of Tet-HER1-CAR-T cells in tumors, which is attributed primarily to the distinct *in vivo* biodistribution behaviors of Doxy@CaCO_3_-PEG and Tet-HER1-CAR-T cells and the pH-responsive release of Doxy by Doxy@CaCO_3_-PEG.

Furthermore, the ELISA results revealed that treatment with Tet-HER1-CAR-T cells and Doxy@CaCO_3_-PEG injections induced much lower serum levels of pro-inflammatory cytokines (e.g. IL-2, TNF-α and IFN-γ) than did treatment with Doxy-activated Tet-HER1-CAR-T cells on Days 2 and 4 (Fig. 3K). These results demonstrate that Doxy@CaCO_3_-PEG-activated Tet-HER1-CAR-T-cell treatment is promising for suppressing nonspecific CAR-T-cell activation-induced systemic secretion of pro-inflammatory cytokines, which is a major safety concern of current CAR-T-cell therapies in the clinic.

### Doxy@CaCO_3_-PEG-activated Tet-HER1-CAR-T-cell therapy effectively suppresses subcutaneous tumors

**Figure 4.**
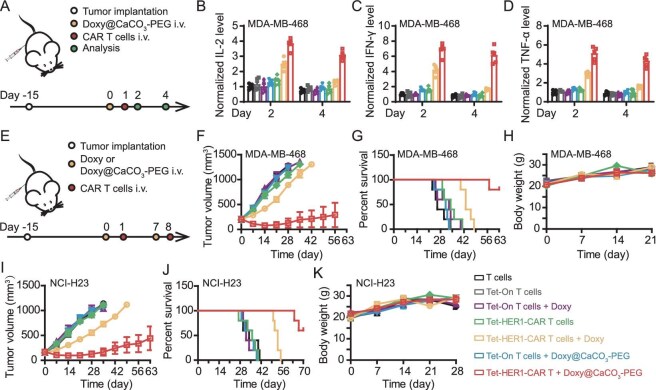
*In vivo* tumor treatment effect of Doxy@CaCO_3_-PEG-activated Tet-HER1-CAR-T-cell therapy in subcutaneous tumor models. (A) Schematic diagram of the experimental schedule for evaluating the intratumoral cytokine expression levels. (B–D) Intratumoral secretion levels of (B) IL-2, (C) IFN-γ and (D) TNF-α in MDA-MB-468-tumor-bearing mice subjected to indicated treatment. (E) Schematic diagram of the experimental schedule for determining the *in vivo* antitumor effect of Doxy@CaCO_3_-PEG-activated Tet-HER1-CAR-T-cell therapy. (F, I) Average tumor growth curves, (G, J) survival rates and (H, K) body weights of MDA-MB-468-tumor-bearing and NCI-H23 tumor-bearing mice subjected to indicated treatment.

We then assessed the therapeutic efficacy of Doxy@CaCO_3_-PEG-activated Tet-HER1-CAR-T cells in both MDA-MB-468- and NCI-H23-tumor-bearing nude mice. By ELISA, the MDA-MB-468 tumors of the mice treated with Doxy@CaCO_3_-PEG and Tet-HER1-CAR-T cells exhibited significant secretion of IL-2, IFN-γ and TNF-α, all of which promoted the cytolysis of tumor cells, at both Days 2 and 4 (Fig. 4A–D). In contrast, free Doxy and Tet-HER1-CAR-T-cell injection led to obvious secretion of these cytokines only on Day 2, further indicating that Doxy@CaCO_3_-PEG can enable the sustained activation of Tet-HER1-CAR-T cells inside tumors. The distinct ability of Doxy@CaCO_3_-PEG and Doxy to activate Tet-HER1-CAR-T cells should be ascribed to their different tumor accumulation efficacy. After that, by recording tumor growth curves and mouse survival rates (Fig. 4E), Doxy@CaCO_3_-PEG-activated Tet-HER1-CAR-T-cell therapy exhibited the most effective tumor-suppression effect and three of the five MDA-MB-468-tumor-bearing mice were fully cured without obvious tumor relapse within 63 days (Fig. 4F and G, and Fig. S15). In contrast, compared with other treatments, free Doxy-activated Tet-HER1-CAR-T-cell therapy only slightly delayed targeted tumor growth. Moreover, the body weights of these treated mice exhibited minimal fluctuations throughout the whole monitoring process (Fig. 4H). The superior tumor-suppression capacity of Doxy@CaCO_3_-PEG-activated Tet-HER1-CAR-T-cell therapy was further confirmed in NCI-H23-lung-tumor-bearing nude mice (Fig. 4I–K and Fig. S16).

Meanwhile, Doxy@CaCO_3_-PEG-activated Tet-HER1-CAR-T exhibited better antitumor efficacy than conventional HER1-CAR-T cells, though Doxy@CaCO_3_-PEG itself exhibited a minimal tumor-suppression effect (Fig. S17A). In contrast, Doxy@CaCO_3_-PEG only slightly enhanced the tumor-suppression effect of conventional HER1-CAR-T cells. Notably, we found that the treatment with two injections of Doxy@CaCO_3_-PEG and Tet-HER1-CAR-T cells exhibited more effective tumor suppression than that with two Doxy@CaCO_3_-PEG injections and one Tet-HER1-CAR-T cell injection, probably ascribed to the limited survival time of CAR-T cells after intravenous infusion [25]. ELISA assay results showed that the pro-inflammatory cytokine levels in both the peripheral blood and tumor tissues of MDA-MB-468-tumor-bearing mice with Doxy@CaCO3-PEG-activated Tet-HER1-CAR-T treatment were comparable to those of control mice at 28 days post treatment (Fig. S17B and C). Taken together, these results imply that treatment with sequential injections of Doxy@CaCO_3_-PEG and Tet-HER1-CAR-T cells effectively suppresses the growth of targeted tumors without causing obvious safety concerns.

### Doxy@CaCO_3_-PEG-activated Tet-HER1-CAR-T-cell therapy suppresses lung tumors

**Figure 5.**
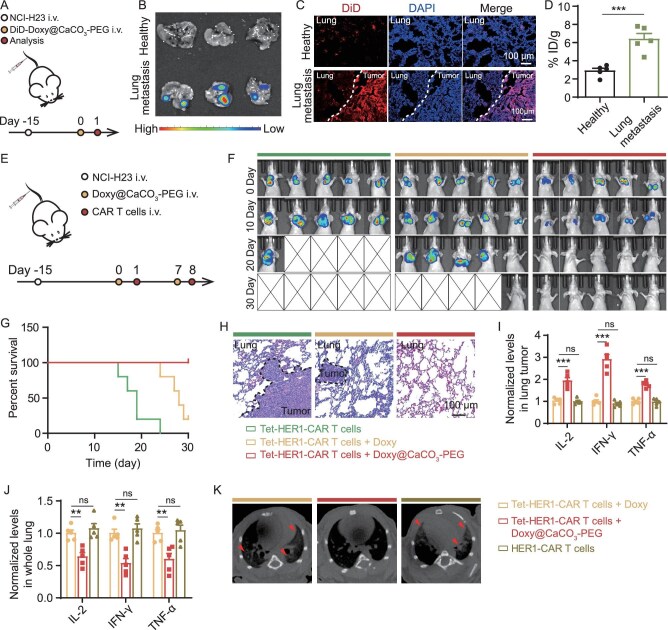
*In vivo* tumor treatment effect of Doxy@CaCO_3_-PEG-activated Tet-HER1-CAR-T-cell therapy against lung metastatic tumors. (A) Schematic diagram of the experimental schedule for evaluating the biodistribution profiles of Doxy@CaCO_3_-PEG in the lungs. (B) *Ex vivo* fluorescence images and (C) representative fluorescence images of lung slices collected from both healthy and NCI-H23 lung-metastasis-bearing mice treated with DiD-Doxy@CaCO_3_-PEG injection and collected at 24 h post injection. (D) Analysis of the amount of Doxy@CaCO_3_-PEG accumulated in the lungs of both healthy and NCI-H23 lung-metastasis-bearing mice at 24 h post injection. (E) Schematic diagram of the experimental schedule for evaluating the ability of Doxy@CaCO_3_-PEG-activated Tet-HER1-CAR-T-cell therapy to suppress lung metastasis. (F) *In vivo* bioluminescence images , (G) survival rates and (H) representative H&E-stained lung slices of luciferase-expressing NCI-H23 lung metastases from mice subjected to indicated treatment. (I‒K) Cytokine expression levels in (I) lung metastatic nodules and (J) whole lungs, and (K) micro-CT images of NCI-H23 lung-metastasis-bearing mice subjected to indicated treatment.

Inspired by the high accumulation efficacy of Tet-HER1-CAR-T cells in the lungs, which are the primary site for both primary lung cancer and secondary metastatic tumors (e.g. TNBC), we investigated the tumor-suppression effect of Doxy@CaCO_3_-PEG-activated Tet-HER1-CAR-T**-**cell therapy in NCI-H23 lung tumor metastasis-bearing nude mice. We first investigated the enrichment potency of DiD-Doxy@CaCO_3_-PEG after intravenous injection in lung tumor metastasis. *Ex vivo* fluorescence imaging revealed that these NCI-H23 metastatic nodules in the lung presented stronger DiD fluorescence signals than did the other regions of the lung without visible metastatic nodules and the lungs of healthy mice at 24 h after the injection of DiD-Doxy@CaCO_3_-PEG (Fig. 5A and B). The superior enrichment capacity of DiD-Doxy@CaCO_3_-PEG in lung metastatic nodules was further confirmed by using confocal microscopy (Fig. 5C). Moreover, by recording the DiD fluorescence of the homogenized lungs collected at 24 h post injection, we found that the amount of Doxy@CaCO_3_-PEG accumulated in the lungs of NCI-H23 tumor lung-metastasis-bearing mice was ∼6.38% ID g^−1^, which was much greater than the ∼2.9% ID g^−1^ observed in healthy mice subjected to the same treatment (Fig. 5D). These results verify that Doxy@CaCO_3_-PEG could also efficiently accumulate in these small metastatic nodules (∼2 mm) after systemic administration.

We then evaluated the efficacy of Doxy@CaCO_3_-PEG-activated Tet-HER1-CAR-T-cell therapy in suppressing the growth of lung metastases in mice bearing luciferase-expressing NCI-H23 lung metastases. To this end, three groups of lung-metastasis-bearing mice received two intravenous injections of Tet-HER1-CAR-T cells (Group I), free Doxy plus Tet-HER1-CAR-T cells (Group II) and Doxy@CaCO_3_-PEG plus Tet-HER1-CAR-T cells (Group III) (Fig. 5E). The *in vivo* bioluminescence imaging results indicated that the bioluminescence signals in the lungs of the mice treated with sequential Doxy@CaCO_3_-PEG and Tet-HER1-CAR-T cell injections gradually decreased and fully disappeared at 12 days after the second injection of Tet-HER1-CAR-T cells (Day 20) (Fig. 5F). In contrast, all the mice injected with the Tet-HER1-CAR-T cells died within 24 days and only one of the five mice treated with free Doxy or the Tet-HER1-CAR-T cells survived (Fig. 5G).

Furthermore, the hematoxylin and eosin (H&E)-staining results indicated that the lung slices of the mice treated with Doxy@CaCO_3_-PEG and Tet-HER1-CAR-T cells presented no obvious tumor nodules on Day 14, whereas the lung slices of the mice subjected to the other two treatments presented obvious tumor nodules (Fig. 5H). Furthermore, the ELISA results revealed that the lung metastatic nodules of the mice treated with Doxy@CaCO_3_-PEG and Tet-HER1-CAR-T cells expressed much greater amounts of pro-inflammatory cytokines (e.g. IL-2, TNF-α and IFN-γ) than those collected from the mice treated with free Doxy plus Tet-HER1-CAR-T cells or conventional HER1-CAR-T cells on Day 10 (Fig. 5I). These results collectively demonstrate that Doxy@CaCO_3_-PEG can effectively activate tumor-infiltrating Tet-HER1-CAR-T cells to suppress the growth of lung metastases.

Considering that Tet-HER1-CAR-T cells have high accumulation efficiency in the lungs, we investigated the safety profile of Doxy@CaCO_3_-PEG-activated Tet-HER1-CAR-T-cell therapy in mice bearing NCI-H23 lung metastases. Through ELISA, we found that the whole-lung lysates of the mice treated with Doxy@CaCO_3_-PEG and Tet-HER1-CAR-T cells presented significantly lower expression levels of IL-2, TNF-α and IFN-γ than did those of the mice treated with Doxy-activated Tet-HER1-CAR-T cells or conventional HER1-CAR-T cells on Day 10 (Fig. 5J). Moreover, the microcomputed tomography (micro-CT) results showed that the lung of mice treated with Doxy-activated Tet-HER1-CAR-T cells or conventional HER1-CAR-T cells exhibited markedly increased brightness on Day 10—a typical imaging feature of pneumonia (Fig. 5K)—which could be induced by high levels of pro-inflammatory cytokines [17]. In sharp contrast, Doxy@CaCO_3_-PEG-activated Tet-HER1-CAR-T-cell therapy exhibited minimal disturbance on the brightness in the lungs of treated mice. These results collectively verify that Doxy@CaCO_3_-PEG-activated Tet-HER1-CAR-T-cell therapy does not cause apparent side effects at the tested dosages because of the spatially selective activation of Tet-HER1-CAR-T cells inside lung metastases.

### Doxy@CaCO_3_-PEG-activated Tet-HER1-CAR-T-cell therapy is a substitution therapy for erlotinib-resistant TNBC

**Figure 6.**
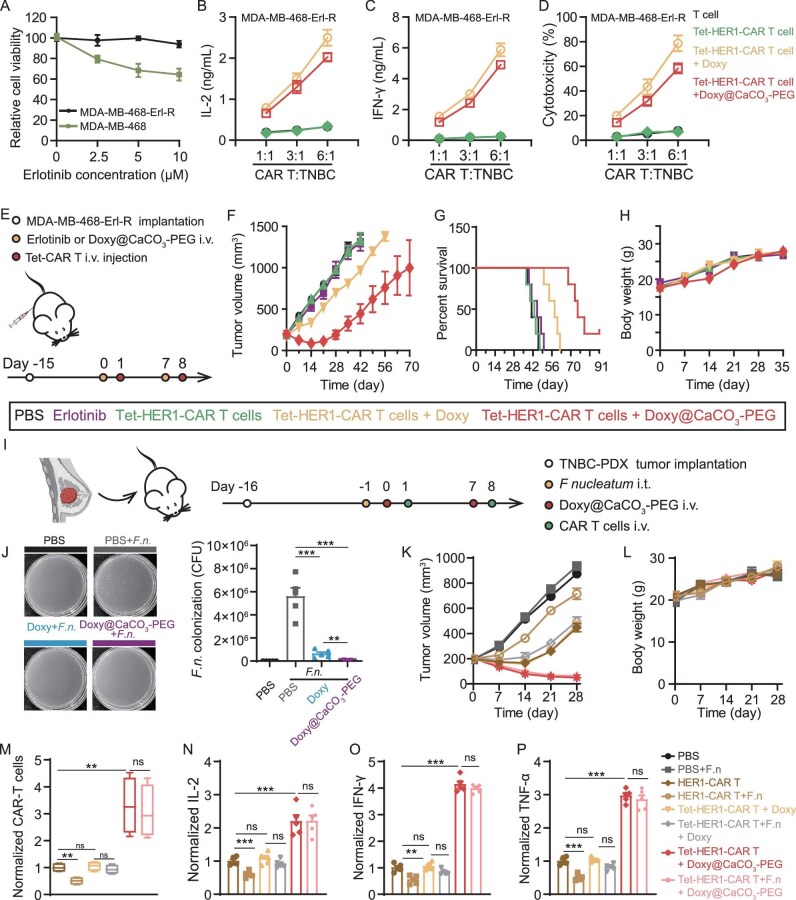
*In vivo* tumor treatment effect of Doxy@CaCO_3_-PEG-activated Tet-HER1-CAR-T-cell therapy against erlotinib-resistant and *F.n.*-colonized TNBC tumors. (A) Relative viability of MDA-MB-468 and MDA-MB-468-Erl-R cells incubated with erlotinib as indicated. (B–D) IL-2 (B) and IFN-γ (C) secretion and cytolysis ability of Tet-HER1-CAR-T cells co-cultured with MDA-MB-468-Erl-R at different ratios and Doxy or Doxy@CaCO_3_-PEG ([Doxy] = 1 μg/mL) for 24 h as indicated. (E) Schematic diagram of the experimental schedule. (F) Average tumor growth curves, (G) survival rates and (H) body weights of erlotinib-resistant MDA-MB-468-tumor-bearing mice subjected to different treatments as indicated. (I) Schematic diagram of the experimental schedule. (J) *F.n.*-colonization profiles of homogenized tumors collected from mice subjected to different treatments as indicated on Day 10. (K) Average tumor growth curves , (L) body weights, (M) intratumoral contents of CAR-T cells and (N‒P) intratumoral cytokine secretion levels of IL-2 in TNBC PDX-bearing mice subjected to indicated treatment. The intratumor CAR-T-cell contents and cytokine release profiles were detected on Day 10. ***P < 0.001, **P < 0.01, * *P* < 0.05.

Erlotinib is a first-line molecular targeted therapy for TNBC and many other HER1-overexpressing cancers that functions by specifically inhibiting the tyrosine kinase activity of HER1 through competitively occupying the adenosine triphosphate (ATP) binding site in the intracellular domains of HER1 [26]. However, clinical evidence has shown that multiple erlotinib treatments might lead to severe acquired resistance in patients through the development of secondary mutations within the drug-binding domain and the activation of compensatory pathways or other pathways [27]. Considering that the extracellular domain of HER1 is typically not changed during the development of acquired resistance [28], we investigated the therapeutic potency of Doxy@CaCO_3_-PEG-activated Tet-HER1-CAR-T-cell therapy against erlotinib-resistant TNBC tumors. Compared with parent MDA-MB-468 cells, erlotinib-resistant MDA-MB-468 cells (MDA-MB-468-Erl-R) established via the drug-concentration-increase method presented comparable surface expression of HER1 but significantly increased tolerance to erlotinib incubation (Fig. 6A and Fig. S18). Furthermore, the cytokine release assay results revealed that the Tet-HER1-CAR-T cells activated by Doxy or Doxy@CaCO_3_-PEG secreted more pro-inflammatory cytokines (e.g. IL-2 and IFN-γ) than did the T cells and Tet-HER1-CAR-T cells when co-cultured with MDA-MB-468-Erl-R cells (Fig. 6B and C). In addition, Tet-HER1-CAR-T cells activated by Doxy or Doxy@CaCO_3_-PEG also showed effective cytolytic potency against co-cultured MDA-MB-468-Erl-R cells (Fig. 6D).

We further explored the therapeutic efficacy of our Doxy@CaCO_3_-PEG-activated Tet-HER1-CAR-T-cell therapy against erlotinib-resistant TNBC tumors in mice. To this end, five groups of MDA-MB-468-Erl-R tumor-bearing mice received i.v. injections of PBS (Group I), erlotinib (Group II), Tet-HER1-CAR-T cells (Group III), Doxy plus Tet-HER1-CAR-T cells (Group IV) and Doxy@CaCO_3_-PEG plus Tet-HER1-CAR-T cells (Group V) twice (Fig. 6E). By recording the tumor growth curves and survival rates, we found that Doxy@CaCO_3_-PEG-activated Tet-HER1-CAR-T-cell therapy had the best suppressive effect on MDA-MB-468-Erl-R tumors, with a mean survival time of 75 days. In marked contrast, Doxy-activated Tet-HER1-CAR-T-cell therapy only slightly suppressed MDA-MB-468-Erl-R tumors and erlotinib treatment alone at the tested dosage had a minimal suppressive effect on tumor growth (Fig. 6F and G and Fig. S19). The body weights of the mice subjected to different treatments were not significantly affected throughout the entire monitoring process (Fig. 6H). These results collectively indicate that Doxy@CaCO_3_-PEG-activated Tet-HER1-CAR-T-cell therapy is a promising substitution treatment strategy for erlotinib-resistant TNBC tumors.

### Doxy@CaCO_3_-PEG-activated Tet-HER1-CAR-T-cell therapy effectively suppresses *F.n.*-colonized TNBC PDXs

Accumulating evidence has demonstrated that *F.n.* infection is a risk factor for the development of colorectal cancer, breast cancer and other types of cancer, and can accelerate tumor progression by suppressing the infiltration and effector functions of tumor-infiltrating lymphocytes (e.g. tumor-killing natural killer cells and CD8^+^ T cells) [29]. Inspired by the superior antibacterial capacity of Doxy, we investigated the therapeutic capacity of Doxy@CaCO_3_-PEG-activated Tet-HER1-CAR-T-cell therapy against *F.n.-*colonized HER1-overexpressing TNBC PDX tumors (Fig. 6I). A plate colony formation assay first revealed that *F.n.* (5 × 10^7^) in the exponential phase upon intratumoral injection colonized these TNBC PDX tumors, whereas the intravenous administration of free Doxy and Doxy@CaCO_3_-PEG effectively suppressed the colonization of *F.n.* in tumors (Fig. 6J).

Then, four groups of mice bearing *F.n.-*colonized HER1-overexpressing TNBC PDX tumors received two injections of PBS, HER1-CAR-T cells, free Doxy plus Tet-HER1-CAR-T cells and Doxy@CaCO_3_-PEG plus Tet-HER1-CAR-T cells, and another four groups of mice bearing HER1-overexpressing TNBC PDX tumors received the same treatments for comparison. Tumor growth curves revealed that intratumoral *F.n.* colonization minimally impaired the growth rate of HER1-overexpressing TNBC PDX tumors, but markedly attenuated the tumor-inhibition effect of conventional HER1-CAR-T-cell therapy (Fig. 6K and Fig. S20). In addition, the body weights of the mice subjected to different treatments were not significantly affected throughout the entire monitoring process (Fig. 6L). Moreover, we found that such *F.n.* colonization also negligibly disrupted the tumor-inhibition effects of both Doxy-activated and Doxy@CaCO_3_-PEG-activated Tet-HER1-CAR-T-cell therapies, the latter of which were the most effective at suppressing tumor growth. Flow cytometry revealed that *F.n.* colonization significantly attenuated the tumor-infiltration efficacy of HER1-CAR-T cells but minimally disrupted the tumor-infiltration efficacy of both Doxy-activated and Doxy@CaCO_3_-PEG-activated Tet-HER1-CAR-T cells (Fig. 6M). Furthermore, the ELISA results indicated that *F.n.* colonization led to significantly reduced secretion levels of pro-inflammatory IL-2, IFN-γ and TNF-α in the tumors of the mice treated with HER1-CAR-T cells, but minimally impaired those of the mice treated with free Doxy-activated and Doxy@CaCO_3_-PEG-activated Tet-HER1-CAR-T cells (Fig. 6N–P). Taken together, these results indicate that Doxy@CaCO_3_-PEG-activated Tet-HER1-CAR-T-cell therapy effectively eradicates *F.n.*-colonized tumors by concurrently killing *F.n.* and neutralizing tumor acidity to increase the tumor-infiltration and effector functions of activated Tet-HER1-CAR-T cells.

## DISCUSSION AND CONCLUSION

CAR-T-cell therapy represents a promising and potent option to selectively eradicate solid tumors, but its clinical translation has been hindered by several drawbacks. Owing to the lack of tumor-specific antigens, CAR-T-cell therapy, including that approved for hematologic malignancies, inevitably causes cytokine release syndrome because of its on-target/off-tumor effects [17,30–35]. To address this issue, we developed a rational strategy to enable the spatially selective activation of engineered Tet-HER1-CAR-T cells for the effective eradication of HER1-positive TNBC tumors by relying on the distinct *in vivo* biodistribution behaviors of T cells and pH-responsive Doxy@CaCO_3_-PEG nanoparticles after systemic administration. Unlike free Doxy, which is able to activate Tet-HER1-CAR-T cells that accumulate in the lungs and liver, Doxy@CaCO_3_-PEG, which can home to tumor sites via enhanced permeability and retention effects and gradually release Doxy in response to tumor acidity, enables the selective activation of Tet-HER1-CAR-T cells in tumors, including small lung metastases. As a result, Doxy@CaCO_3_-PEG-activated Tet-HER1-CAR-T-cell therapy led to less secretion of pro-inflammatory cytokines in blood and normal lung tissues and pneumonia than free Doxy-activated Tet-HER1-CAR-T-cell therapy and conventional HER1-CAR-T-cell therapy. However, due to the limited circulation time of Doxy@CaCO_3_-PEG, which is an unresolved challenge in nanomedicine, and the restricted *in vivo* survival of Tet-HER1-CAR-T cells in mice, our therapeutic regimen required multiple injections of both Doxy@CaCO_3_-PEG and Tet-HER1-CAR-T cells to achieve prolonged CAR-T activation and sustained antitumor effects.

The hostile tumor microenvironment is a major cause of current failure of CAR-T-cell therapy for solid tumors because it restricts the tumor-infiltration and effector functions of CAR-T cells [36]. Owing to the superior ability of CaCO_3_ to neutralize protons, CaCO_3_-based formulation has been confirmed to be capable of markedly enhancing the tumor-suppression effect of HER1-CAR-T cells via protecting them from tumor acidity-induced dysfunctions, such as reduced cytokine secretion and cytolysis capacity [5]. However, Doxy@CaCO_3_-PEG at current dosages only slightly enhanced the tumor-suppression effect of conventional HER1-CAR-T-cell therapy, likely due to non-optimized dosing. This suggests that the therapeutic efficacy of Doxy@CaCO_3_-PEG-activated Tet-HER1-CAR-T cell therapy could be further improved by adjusting their injection dose, frequency and/or sequence. In addition, owing to the intrinsic antibacterial capacity of Doxy, Doxy@CaCO_3_-PEG could also suppress the intratumoral colonization of *F.n.*, which can promote the development of breast cancer and other solid tumors, and disable diverse cancer treatments by driving tumor immunosuppression [29]. Therefore, Doxy@CaCO_3_-PEG-activated Tet-HER1-CAR-T-cell therapy markedly improved tumor suppression in different HER1-overexpressing TNBC tumor models compared with free Doxy-activated Tet-HER1-CAR-T-cell therapy and conventional HER1-CAR-T-cell therapy. In addition, erlotinib is a first-line molecular targeted therapy for HER1-overexpressing tumors, but is limited by a high frequency of acquired drug resistance in real-world clinical practice. Owing to its unique tumor-eradicating mechanism, Doxy@CaCO_3_-PEG-activated Tet-HER1-CAR-T-cell therapy was also able to effectively suppress the growth of erlotinib-resistant TNBC tumors in mice.

In summary, we demonstrate that the as-prepared pH-responsive Doxy@CaCO_3_-PEG is able to enable spatially selective activation of engineered Tet-HER1-CAR-T cells via the concurrent tumor-localized delivery and pH-responsive release of Doxy and neutralization of tumor acidity. Together with its excellent ability to neutralize tumor acidity and eradicate immunosuppressive *F.n.*, Doxy@CaCO_3_-PEG can synergize with such Tet-HER1-CAR-T cells to selectively eradicate HER1-overexpressing solid tumors and significantly reduce safety risks. Although PEGylated liposomal nanocarriers are reported to cause unwanted immune responses [37], thereby impairing the tumor accumulation of Doxy@CaCO_3_-PEG, this could be resolved by developing a new generation of anti-fouling polymers [38–40]. Together with the superior biocompatibility of CaCO_3_ and liposomes, Doxy@CaCO_3_-PEG-activated Tet-HER1-CAR-T-cell therapy holds great promise for future clinical translation.

## MATERIALS AND METHODS

Nanjing Normal University's laboratory animal center approved all the animal experiments. The excised HER1-overexpressing TNBC masses were obtained from patients at the First Affiliated Hospital, and a letter of authorization to construct PDX mouse models was signed.

Detailed descriptions of materials and methods are available as supplementary data at NSR online.

## Supplementary Material

nwaf306_Supplemental_File
