# Supplementary material for: pH-responsive activation of Tet-On inducible CAR-T cells enables spatially selective treatment of targeted solid tumors at reduced safety risk
Source: Natl Sci Rev. 2025 Jul 31;12(9):nwaf306. doi: 10.1093/nsr/nwaf306 (PMC12416280; doi:10.1093/nsr/nwaf306)
Supplement: nwaf306_Supplemental_File [file nwaf306_supplemental_file.pdf]

pH-responsive activation of Tet-On inducible CAR-T cells enables spatially selective treatment of targeted solid tumors at reduced safety risk

Yan Liu[1,#,*], Yu Hao[2,#], Jin Zhang[1], Mengmeng Zhang[1], Jiahui Chen[1], Zhengmiao Xia[1], Minming Chen[2], Xiang Lv[1], Xinxing Ma[3], Yehui Zhou[4], Jing Xu[5], Linqi Zhu[6], Wei Zhou[6], Liangzhu Feng[2,6,*]

[1]Jiangsu Key Laboratory for Molecular and Medical Biotechnology, Cancer Institute, Department of Biochemistry, College of Life Science, Nanjing Normal University, Nanjing 210023, China;

[2]Institute of Functional Nano & Soft Materials (FUNSOM), Jiangsu Key Laboratory for Carbon-Based Functional Materials & Devices, Soochow University, Suzhou 215123, China;

[3]Department of Radiology, The First Affiliated Hospital of Soochow University, Suzhou 215006, China;

[4]Department of General Surgery, The First Affiliated Hospital of Soochow University, Suzhou 215000, China;

[5]Department of Clinical Laboratory, Zhongda Hospital Southeast University, Nanjing 210009, China;

[6]Department of Clinical Laboratory, The Third Affiliated Hospital of Soochow University, Changzhou 213003, China

*__Corresponding authors__. E-mails: lllIiuyan@sina.com; lzfeng@suda.edu.cn

[#]Equally contributed to this work

METHOD

Chemicals and reagents

Calcium chloride anhydrous ($CaCl_2$) was obtained from Sinopharm Chemical Reagent Co., Ltd., China. Ammonia bicarbonate (NH_4HCO_3) was obtained from Shanghai Macklin Biochemical Co., Ltd. 1,2-DDioleoyl-sn-glycero-3-phosphate (sodium salt) (DOPA) was obtained from Avanti Lipids Polar, Inc. 1,2-DDihexadecanoyl-sn-glycero-3-phosphocholine (DPPC), 1,2-distearoyl-snglycero-3-phosphoethanolamine-N-[methoxy(polyethylene glycol)-

5000] (DSPE-PEG_{5k}) and cholesterol were purchased from Xi'an Ruixi Biological Technology Co., Ltd. Doxycycline (Doxy) was purchased from Sigma–Aldrich. Anti-CD3ζ, anti-HER1, and anti-β-actin antibodies were obtained from Cell Signaling Technology. The anti‑mouse and anti‑rabbit immunoglobin G (IgG) secondary antibodies were obtained from Santa Cruz Biotechnology. ELISA kits were obtained from Dakewe.

Synthesis and characterization of Doxy@$CaCO_3$-PEG

Amorphous $CaCO_3$ nanoparticles were synthesized according to our previous work. To synthesize Doxy@$CaCO_3$ nanoparticles, 12 μL of Doxy solution (20 mg mL^{-1}) was added to 1 mL of $CaCO_3$ solution (4 mg mL^{-1} in ethanol) and then stirred at room temperature for 24 h. After that, Doxy@$CaCO_3$ nanoparticles were collected and washed with ethanol by centrifugation at 14,800 rpm. The obtained Doxy@$CaCO_3$ nanoparticles were subsequently coated with DOPA, DPPC, cholesterol and DSPE-PEG_{5k} according to our previously used methods. The Doxy@$CaCO_3$-PEG obtained after purification via centrifugation was stored at 4°C for further experiments.

The morphologies of the $CaCO_3$ and Doxy@$CaCO_3$ nanoparticles were recorded via TEM imaging (TALOS 200X, Sigma). The size distribution profiles of the Doxy@$CaCO_3$-PEG nanoparticles incubated with various physiological solutions (H_2O, PBS and FBS) at different pH values (7.4, 6.5, and 5.5) were measured via a dynamic light scattering (DLS) zetasizer (Nano ZS90, Malvern, USA). The UV–vis–NIR spectra of free Doxy and Doxy@$CaCO_3$-PEG were acquired by using a UV–Vis–NIR spectrophotometer (GENESYS 10S, Thermo Scientific).

pH-responsive release of Doxy and the proton neutralization profiles of Doxy@$CaCO_3$-PEG

To evaluate the proton neutralization ability of Doxy@$CaCO_3$-PEG, 20 μL of Doxy@$CaCO_3$-PEG (the concentration of $CaCO_3$) was added to an acidic solution of lactic acid supplemented Dulbecco's phosphate-buffered saline (DPBS, [lactic acid] = 2 mM, pH ~6.5), incubated at room temperature for 1 h before being mixed with BCECF (10 μM) and imaged with an IVIS Lumina III *in vivo* fluorescence imaging system (PerkinElmer) (ex. 440 nm, em. 535 nm; ex. 480 nm, em. 535 nm).

To evaluate the pH-dependent release behavior of Doxy from Doxy@$CaCO_3$-PEG, Doxy@$CaCO_3$-PEG was dissolved in PBS at pH 5.5, 6.5, or 7.4 and then incubated at 37 ℃. At various time points, the released Doxy was collected via ultrafiltration via an Amicon Ultra Centrifugal Filter with a molecular weight cutoff (MWCO) of 100 kDa. The absorbance of Doxy was recorded at 360 nm via a microplate reader.

Cell experiments

MDA-MB-468 and NCI-H23 cells were purchased from ATCC. MDA-MB-468-Erl-R was constructed via the drug concentration increment method in our laboratory. Both MDA-MB-468 and MDA-MB-468-Erl-R cells were cultured in Dulbecco's modified Eagle's medium (DMEM) supplemented with 10% fetal bovine serum and 1% penicillin–streptomycin solution in a humidified atmosphere containing 5% CO_2 at 37 ℃. NCI-H23 cells were cultured in Roswell Park Memorial Institute-1640 medium supplemented with 10% fetal bovine serum and 1% penicillin–streptomycin solution in a humidified atmosphere containing 5% CO_2 at 37 ℃.

T cells were first isolated from the peripheral blood mononuclear cells of healthy donors and then activated and cultured in T-cell expansion medium supplemented with anti-CD3/CD28 beads, IL-2, 10% human serum, and 1% penicillin–streptomycin in a humidified atmosphere containing 5% CO_2 at 37 ℃. To construct the Tet-HER1-CAR-T cells, the anti-HER1-specific single‑chain variable fragment and third‑generation CAR fragment containing CD8a, CD8, CD28TM, CD28, 4-1BB, and CD3ζ were cloned and inserted into the Tet-On vector to obtain the fused Tet-HER1-CAR expression sequence. T cells were subsequently transduced with lentiviral vectors containing rtTA and Tet-On or Tet-HER1-CAR to generate Tet-On T cells or Tet-HER1-CAR-T cells, respectively. Then, the obtained Tet-On T cells and Tet-HER1-CAR-T cells were separately incubated with Doxy or Doxy@$CaCO_3$-PEG ([Doxy] = 10^1-10^7 pg/mL) for 0-48 h to evaluate the inducible expression profiles of the HER1-CAR by recording the expression of EGFP and CD3ζ via flow cytometry, RT–PCR, and western blotting, respectively. Doxy or Doxy@$CaCO_3$-PEG was removed from the medium of activated Tet-HER1-CAR-T cells for 0-48 h, and the deactivation of HER1-CAR-T cells was evaluated by recording the expression of EGFP via

flow cytometry.

To evaluate the viability of the cells, T cells, Tet-On T cells, and Tet-HER1-CAR-T cells prepared in 96-well plates at a density of 5×10^4 cells per well were cultured at pH 7.4 or pH 6.5 (lactic acid) for the indicated times. The MTT method was subsequently used to detect cell viability according to the manufacturer's procedure. MDA-MB-468 and NCI-H23 cells preseeded in 96-well plates at a density of 5×10^4 cells per well were cultured with Doxy or Doxy@$CaCO_3$-PEG (Doxy=1 μg/mL) for 24 h before their viability was evaluated via the Cell Counting Kit-8 (CCK-8) method.

To evaluate the cytokine release and cytolysis abilities of Tet-HER1-CAR-T cells toward targeted cancer cells, MDA-MB-468 and NCI-H23 cells preseeded in a 96-well plate at a density of 1×10^4 cells per well were cocultured with a suitable density of Tet-HER1-CAR-T cells or other T cells with Doxy or Doxy@$CaCO_3$-PEG (Doxy=1 μg/mL) at pH 7.4 or pH 6.5 for 24 h. An enzyme - linked immunosorbent assay was subsequently used to detect the IL-2, IFN-γ, and TNF-α concentrations in the supernatants according to the manufacturer's procedures. A lactate dehydrogenase assay was used to detect the cytolysis ability of the Tet-HER1-CAR-T cells according to the manufacturer's procedures.

To evaluate the ability of low-pH conditions to inhibit the STAT5-ERK axis in CAR-T cells, Tet-On-T cells and Tet-HER1-CAR-T cells seeded in 6-well plates were cultured with Doxy or Doxy@$CaCO_3$-PEG (Doxy=1 μg/mL) at pH 7.4 (normal condition) or pH 6.5 (lactic acid) for 24 h. Western blotting was subsequently used to detect the intracellular expression of p-STAT5 and p-ERK.

To evaluate erlotinib resistance in cancer cells, MDA-MB-468 cells and MDA-MB-468-Erl-R cells preseeded in 96-well plates at a density of 1×10^4 cells per well were cultured with erlotinib (2.5–10 μM) for 24 h. The CCK-8 method was subsequently used to detect cell viability as described above.

Animal experiments

Four-week-old BALB/c nude mice and NSG mice were purchased from Nanjing University's Model Animal Research Center. All the animal experiments were approved by Nanjing Normal University's laboratory animal center. To construct a subcutaneous tumor model,

MDA-MB-468 and NCI-H23 cells (5×10^6 cells) suspended in 100 μL of PBS were subcutaneously injected into nude mice. To construct a subcutaneous erlotinib-resistant TNBC tumor model, MDA-MB-468-Erl-R cells (5×10^6 cells) were injected into the mammary fat pads of BALB/c nude mice. HER1-overexpressing TNBC masses were obtained from patients at the First Affiliated Hospital and signed a letter of authorization. Then, tumor cells (5×10^6 cells) were injected into the mammary fat pads of NSG mice. To construct a lung tumor metastatic mouse model, NCI-H23 cells (5×10^6 cells) were intravenously injected into BALB/c nude mice.

To evaluate the blood circulation profile of Doxy@$CaCO_3$-PEG, Doxy@$CaCO_3$-PEG noncovalently labeled with hydrophobic DiD was subcutaneously injected into healthy BALB/c nude mice. Then, whole-blood samples from the mice were withdrawn at different time intervals after the injections and homogenized to measure the fluorescence intensity of DiD via a fluorometer.

To evaluate the biodistribution profile of Doxy@$CaCO_3$-PEG, DiD-labeled Doxy@$CaCO_3$-PEG was intravenously injected into MDA-MB-468/NCI-H23 tumor-bearing BALB/c nude mice. Twenty-four hours later, the main tissues and tumors of the injected mice were collected and homogenized to measure the fluorescence intensity of DiD via a fluorometer.

To evaluate the proton neutralization capacity of $CaCO_3$, MDA-MB-468/NCI-H23 tumor-bearing BALB/c nude mice were intravenously injected with PBS, Doxy, Doxy + $CaCO_3$-PEG, or Doxy@$CaCO_3$-PEG (Doxy = 1 mg/kg, $CaCO_3$ = 30 mg/kg). The pH values of the tumors in the mice before and at 24 h after the different treatments were detected by using an invasive pH microelectrode.

To evaluate the tumor infiltration and activation profiles of Tet-HER1-CAR-T cells in targeted tumors and normal organs, Doxy or Doxy@$CaCO_3$-PEG (Doxy=1 mg/kg) was injected into MDA-MB-468/NCI-H23 tumor-bearing BALB/c nude mice via the tail vein at 0 days. On day 1, Tet-HER1-CAR-T cells (5×10^6 cells) and other T cells were injected via the tail vein. On days 2 and 4, the tumors or organs were collected to analyze the expression of RFP or EGFP via real-time PCR and immunofluorescence.

To evaluate the cytokine release profiles, MDA-MB-468/NCI-H23 tumor-bearing mice subjected to the same treatments described above were sacrificed at 24 h after the injection of CAR-T cells, and their tumors or whole blood samples were collected to measure the expression of IL-2, IFN-γ, and TNF-α by corresponding ELISA kits according to the manufacturer's instructions.

To evaluate the tumor suppression effect of Doxy@$CaCO_3$-PEG-activated Tet-HER1-CAR-T cells *in vivo,* Doxy or Doxy@$CaCO_3$-PEG (Doxy = 1 mg/kg) was intravenously injected into MDA-MB-468/NCI-H23 tumor-bearing BALB/c nude mice twice on days 0 and 7. Twenty-four hours after each injection, Tet-HER1-CAR-T cells (5×10^6) or other T cells were injected via the tail vein. A digital caliper was used to measure the tumor length (L) and width (W) of each mouse to calculate the tumor volume (V) via the formula $V = 1/2*L*W^2$. A digital balance was used to measure body weight during the monitoring process.

To evaluate the distribution profile of Doxy@$CaCO_3$-PEG in lung tumor metastasis and normal lung tissues, DiD-labeled Doxy@$CaCO_3$-PEG (Doxy = 1 mg/kg) was first injected into healthy BALB/c nude mice or NCI-H23 lung metastasis-bearing BALB/c nude mice via the tail vein. Twenty-four hours later, the lungs of the injected mice were collected to measure their DiD fluorescence intensity via an IVIS Lumina III *in vivo* fluorescence imaging system and then sliced or homogenized to measure the fluorescence intensity of DiD via a fluorescence confocal microscope or fluorometer, respectively.

To evaluate the therapeutic potency of Doxy@$CaCO_3$-PEG-activated Tet-HER1-CAR-T cells against lung tumor metastasis, PBS, Doxy or Doxy@$CaCO_3$-PEG (Doxy = 1 mg/kg) was injected into NCI-H23 lung metastases from BALB/c nude mice twice on days 0 and 7 via the tail vein. Twenty-four hours after each injection, Tet-HER1-CAR-T cells (5×10^6) were injected via the tail vein. *In vivo* bioluminescence imaging was used to monitor the growth of NCI-H23 lung metastases, which were inoculated with luciferase-expressing NCI-H23 cells. Another batch of NCI-H23 lung metastasis-bearing mice that received two treatments with Doxy-activated Tet-HER1-CAR-T cells, Doxy@$CaCO_3$-PEG-activated Tet-HER1-CAR-T cells or conventional HER1-CAR-T cells were subjected to micro-CT to evaluate the symptoms of pneumonia in the lungs at 10 days. The metastatic nodules and whole lungs

were homogenized to record the intensity of DID as mentioned above, and the whole lungs were also sliced for H&E staining on day 14.

To evaluate the therapeutic potency of Doxy@$CaCO_3$-PEG-activated Tet-HER1-CAR-T cells against erlotinib-resistant MDA-MB-468 tumors, erlotinib (50 mg/kg), Doxy or Doxy@$CaCO_3$-PEG (Doxy = 1 mg/kg) was injected into the tumor-bearing BALB/c nude mice twice at days 0 and 7 via the tail vein. Twenty-four hours after each injection, Tet-HER1-CAR-T cells (5×10^6) were injected via the tail vein. The tumor volumes and body weights of the treated mice were measured as described above.

To evaluate the therapeutic potency of Doxy@$CaCO_3$-PEG-activated Tet-HER1-CAR-T cells against *F.n.*-colonized TNBC PDX tumors in NSG mice, *F.n.* (5×10^7) was intratumorally injected into each TNBC PDX tumor-bearing mouse at -1 day. Doxy or Doxy@$CaCO_3$-PEG (Doxy = 1 mg/kg) was injected twice via the tail vein at days 0 and 7. On days 1 and 8, Tet-HER1-CAR-T cells or HER1-CAR-T cells (5×10^6) were also injected twice via the tail vein. The tumor volumes and body weights of the treated mice were measured as described above. At 10 days, the tumors of treated mice were collected to determine the degree of *F.n.* colonization via the standard bacterial colony formation assay. The tumor infiltration, activation and cytokine release abilities of the Tet-HER1-CAR-T cells were evaluated via RT–PCR and ELISA, respectively, as previously described.

Statistical analysis

GraphPad Prism 8.02 software was used for statistical analysis. The data are presented as the means ± standard errors of the means of at least three independent experiments, and *t* tests were used for statistical quantification.

Schematic illustrations

Schematic illustrations were created with Biorender.com and Adobe Illustrator.

Supplementary Figures:

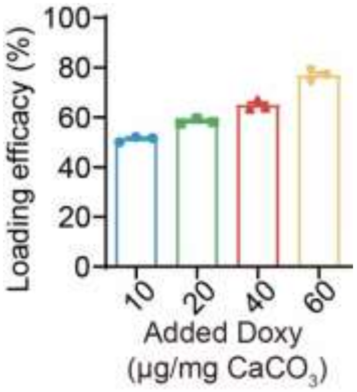

Figure S1. Loading efficiency of Doxy in Doxy@$CaCO_3$.

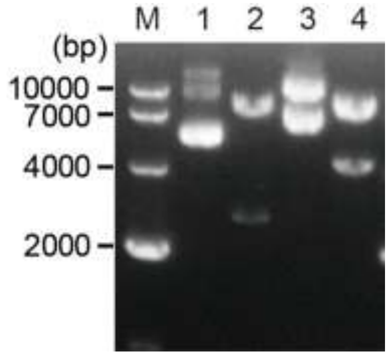

Figure S2. Agarose electrophoresis results. M: DNA marker; Lane 1, Tet-ON plasmid; Lane 2, *NdeI/PspXI* - treated Tet-On DNA products; Lane 3, Tet-HER1-CAR plasmid; Lane 4, *NdeI/PspXI* - treated Tet-HER1-CAR DNA products.

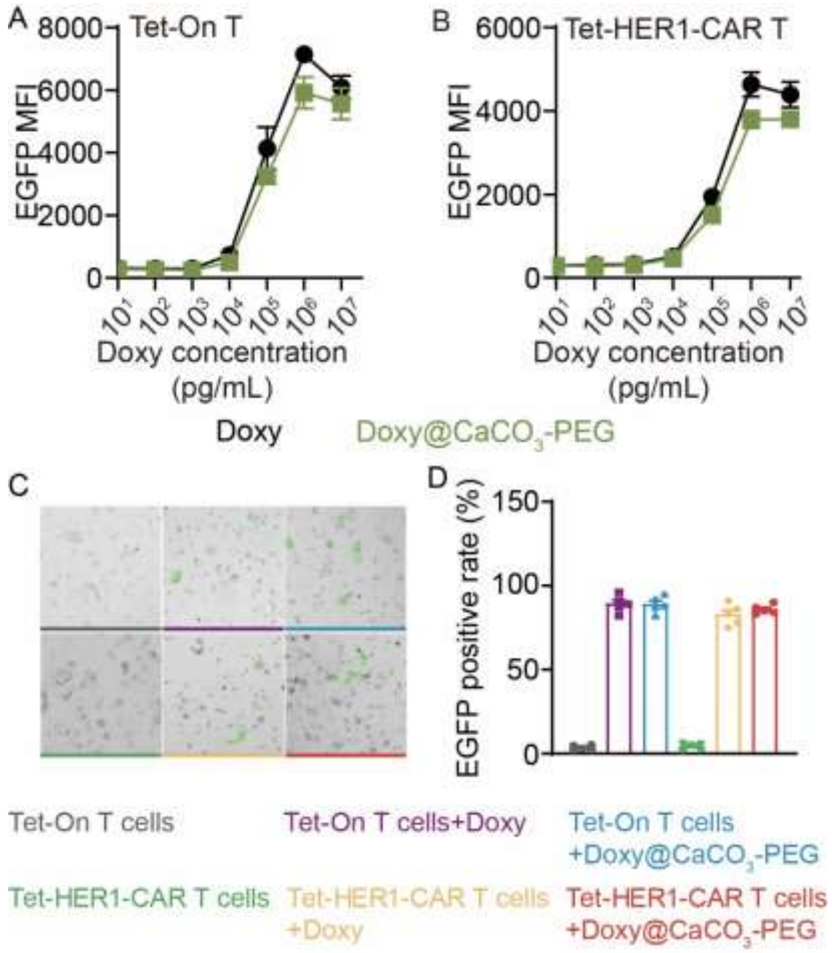

Figure S3. Doxy@$CaCO_3$-PEG enables activation of Tet-HER1-CAR T cells.
Flow cytometric analysis of EGFP expression in Tet-On T cells (**A**) and Tet-HER1-CAR T cells (**B**) incubated with Doxy and Doxy@$CaCO_3$-PEG at different concentrations of Doxy for 24 h. (**C**) Representative images showing that Tet-On T cells, Tet-HER1-CAR T cells activated by Doxy, or Doxy@$CaCO_3$-PEG. (**D**) Flow cytometry analysis of the percentages of activated Tet-On T cells and Tet-HER1-CAR T cells.

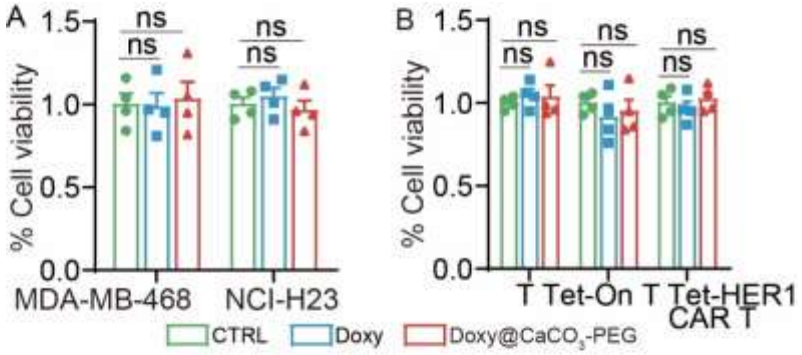

Figure S4. Cytotoxicity behaviors of Doxy@$CaCO_3$-PEG.
(**A**) Relative cell viability of MDA-MB-468 and NCI-H23 cells treated with PBS, Doxy, or Doxy@$CaCO_3$-PEG for 24 h. (**B**) Relative cell viability of T cells, Tet-On T cells and Tet-

HER1-CAR T cells treated with PBS, Doxy, or Doxy@$CaCO_3$-PEG for 24 h. The concentrations of Doxy = 1 μg/mL.

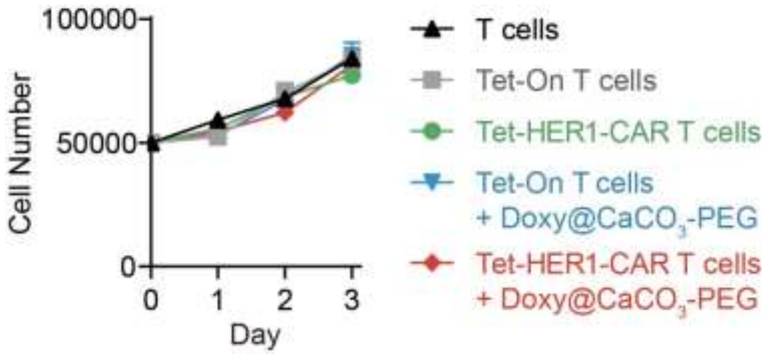

Figure S5. Cell proliferation behavior of different T cells.
Cell proliferation behaviors of T cells, Tet-On T cells, and Tet-HER1-CAR T cells in the presence and absence of Doxy@$CaCO_3$-PEG (Doxy = 1 μg/mL).

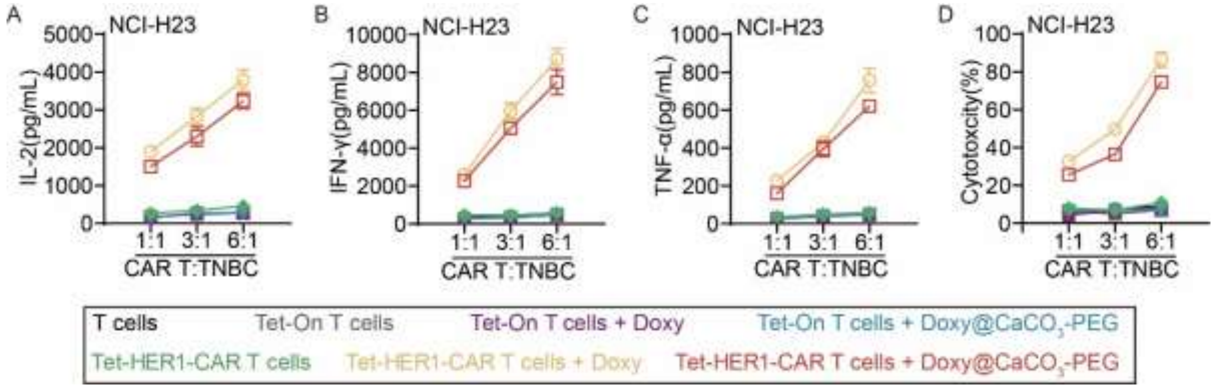

Figure S6. Cytokine release and cytolysis ability of activated Tet-HER1-CAR T cells.
(A-C) The secretion levels of IL-2 (**A**), IFN-γ(**B**), and TNF-α (**C**) of Tet-HER1-CAR T cells or other T cells cocultured with NCI-H23 cells at different feeding ratios and Doxy or Doxy@$CaCO_3$-PEG ([Doxy] = 1 μg/mL) for 24 h as indicated. (**D**) Cytolysis ability of Tet-HER1-CAR T cells or other T cells against cocultured NCI-H23 cells in the presence of Doxy@$CaCO_3$-PEG ([Doxy] = 1 μg/mL) for 24 h.

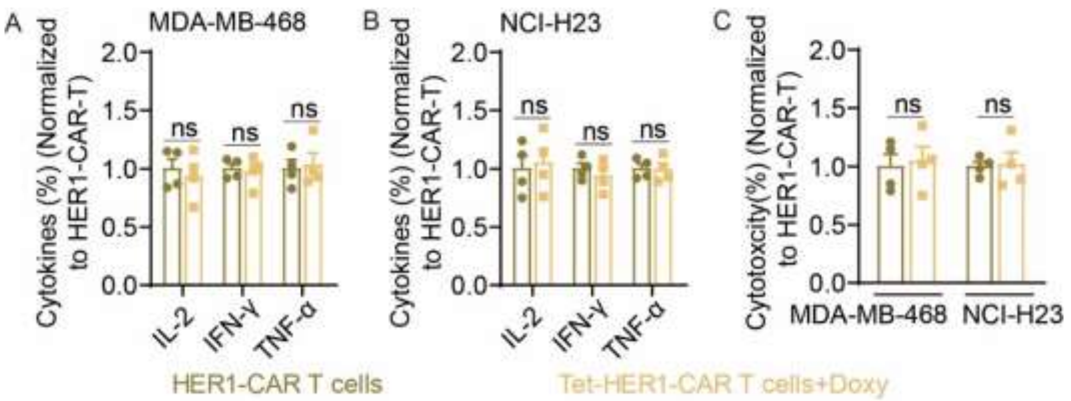

Figure S7. Cytokine secretion and cytolysis ability of HER1-CAR T cells and Doxy activated Tet-HER1-CAR T cells.

(A, B) Cytokine release profiles of HER1-CAR T cells and Doxy activated Tet-HER1-CAR T cells cocultured with MDA-MB-468 cells (**A**) or NCI-H23 cells (**B**) at a feeding ratio of 1:1 ratio for 24 h. (**C**) A standard LDH release assay was used to detect the cytolysis capacity of HER1-CAR T cells and Doxy activated Tet-HER1-CAR T cells in the presence of MDA-MB-468 cells or NCI-H23 cells.

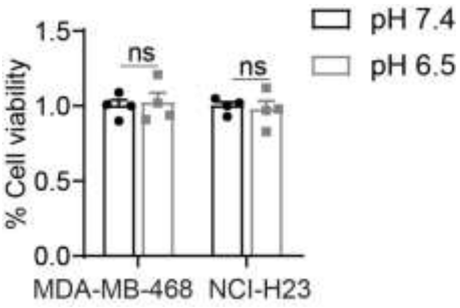

Figure S8. Relative viability of MDA-MB-468 and NCI-H23 cells incubated at indicated pHs for 24 h.

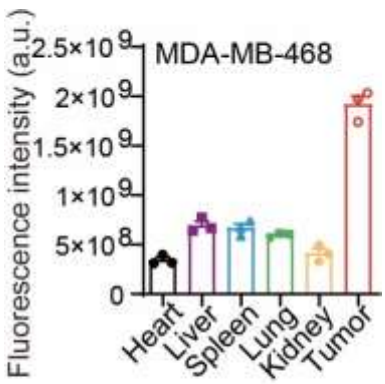

Figure S9. Semiquantitative analysis of DID fluorescence intensity in MDA-MB-468 tumors or main organs based on the fluorescence images shown in Figure 3C.

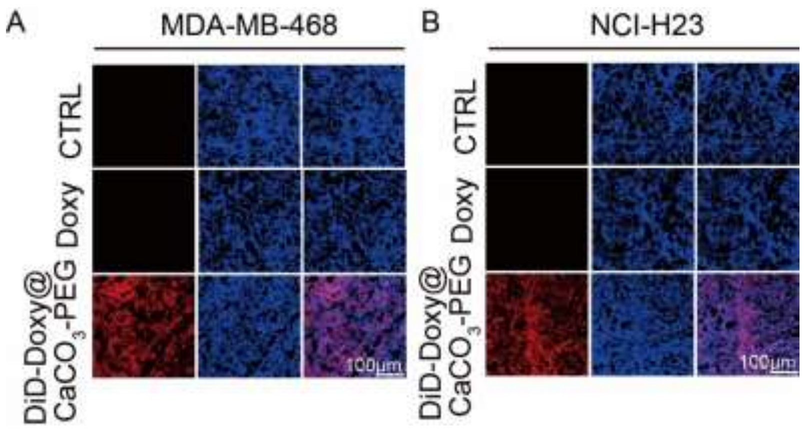

Figure S10. The enrichment capacity of Doxy@$CaCO_3$-PEG in tumors.
Representative fluorescence images of tumor slices of (**A**) MDA-MB-468 and (**B**) NCI-H23 tumor-bearing mice with different treatments as indicated.

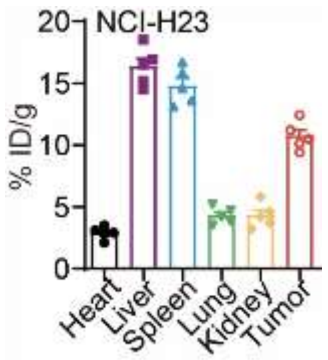

Figure S11. Biodistribution profiles of DiD-Doxy@$CaCO_3$-PEG in NCI-H23 tumor-bearing mice.

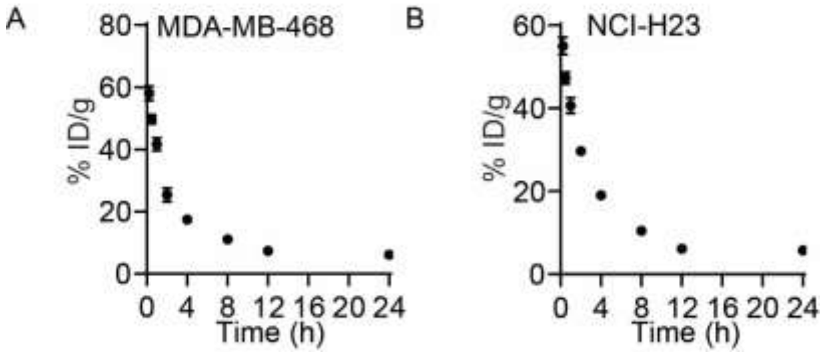

Figure S12. Blood circulation profiles of DiD-Doxy@$CaCO_3$-PEG in MDA-MB-468 (**A**) and NCI-H23 (**B**) tumor-bearing mice determined by recording the DiD fluorescence intensity.

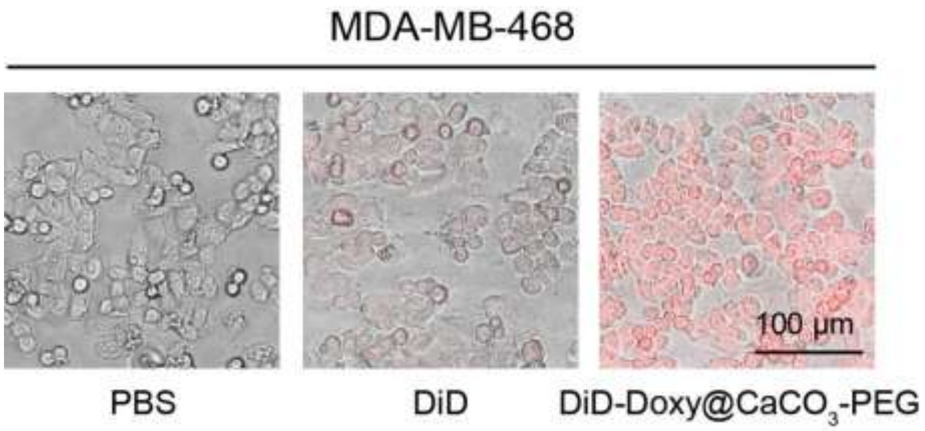

Figure S13. Fluorescence images of MDA-MB-468 cells incubated with PBS, DiD, and DiD-Doxy@$CaCO_3$-PEG for 24 h.

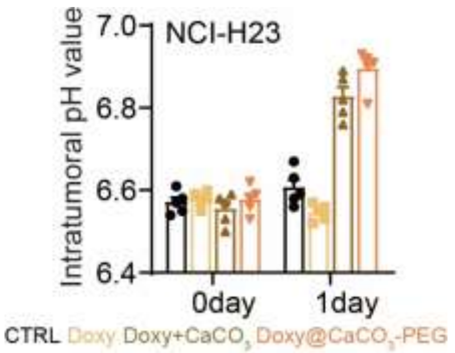

Figure S14. Intratumoral pH values of the NCI-H23 tumor bearing mice recorded on day 0 and 1 day after the treatments of Doxy@$CaCO_3$-PEG, Doxy+$CaCO_3$, Doxy and PBS.

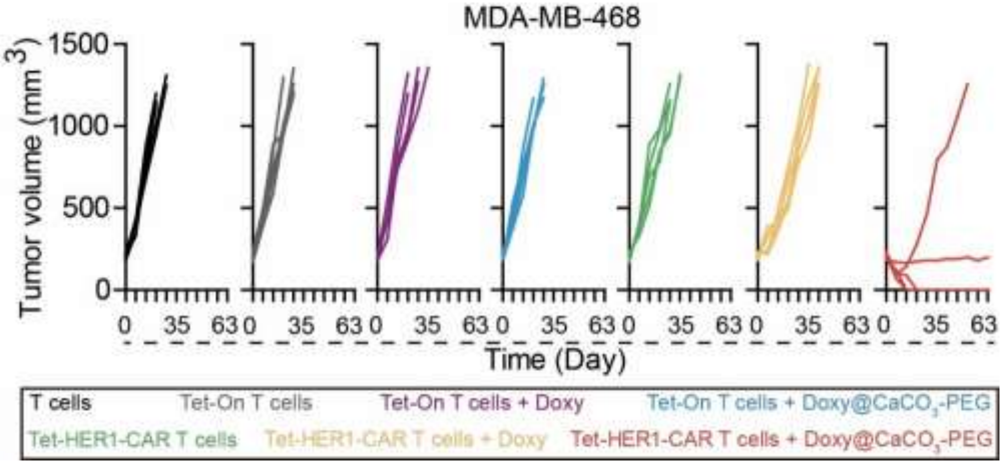

Figure S15. Individual tumor growth curves of MDA-MB-468 tumors in mice with different treatments as indicated.

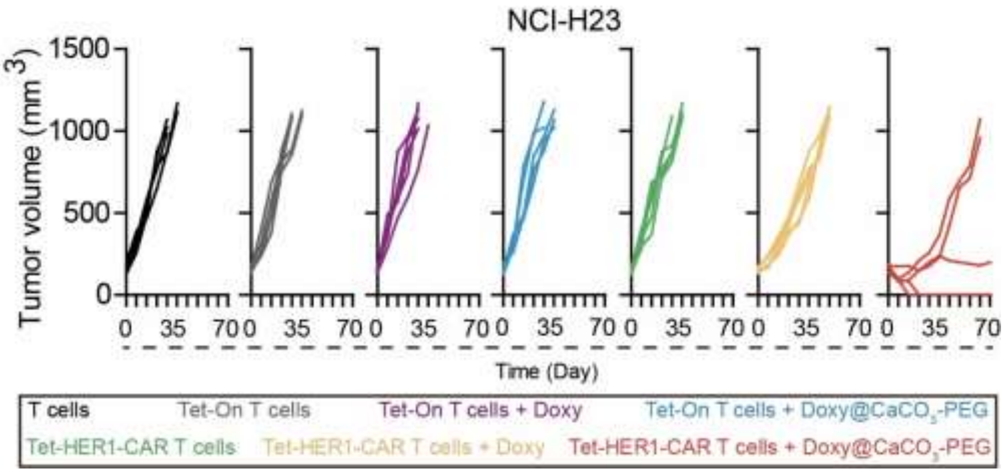

Figure S16. Individual tumor growth curves of NCI-H23 tumors in mice with different treatments as indicated.

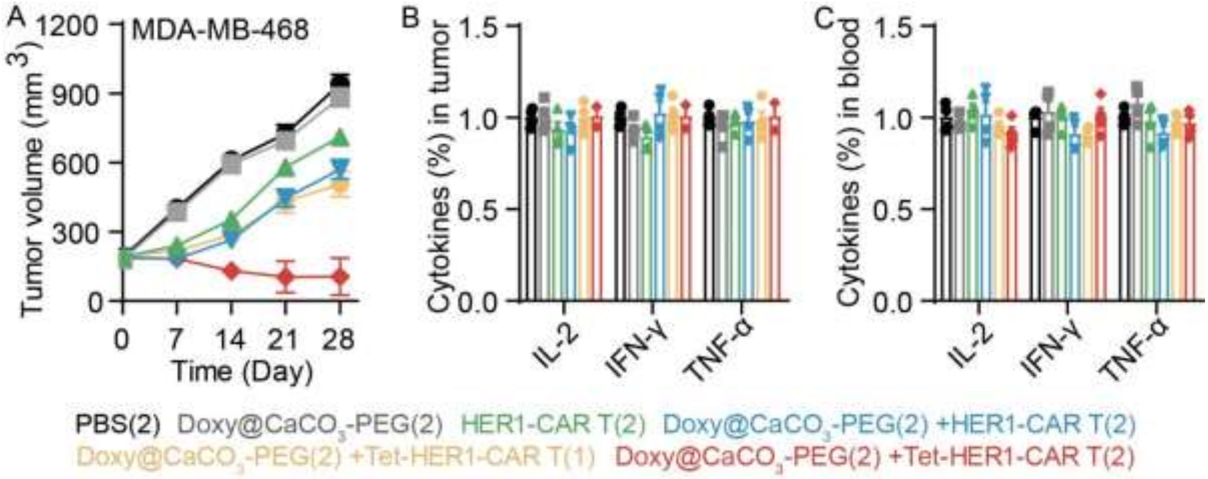

Figure S17. *In vivo* tumor suppression and safety profiles of Doxy@$CaCO_3$-PEG-activated Tet-HER1-CAR T-cell therapy in MDA-MB-468 tumor model. (**A**) Average tumor growth curves of MDA-MB-468 tumor-bearing mice subjected to indicated treatments. (**B**) Relative intratumoral cytokine levels and (**C**) relative serum cytokine levels in MDA-MB-468 tumor-bearing mice after different treatments as indicated.

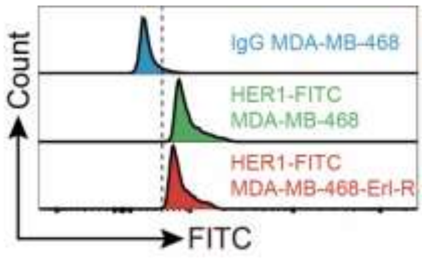

Figure S18. HER1 expression in MDA-MB-468 and MDA-MB-468-Erl-R cells detected by flow cytometry.

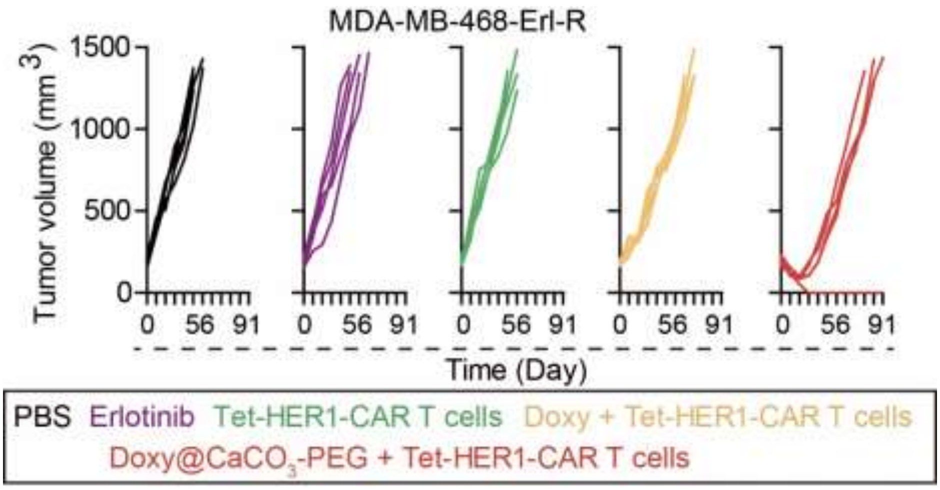

Figure S19. Individual tumor growth curves of MDA-MB-468-Erl-R tumors in mice with different treatments as indicated.

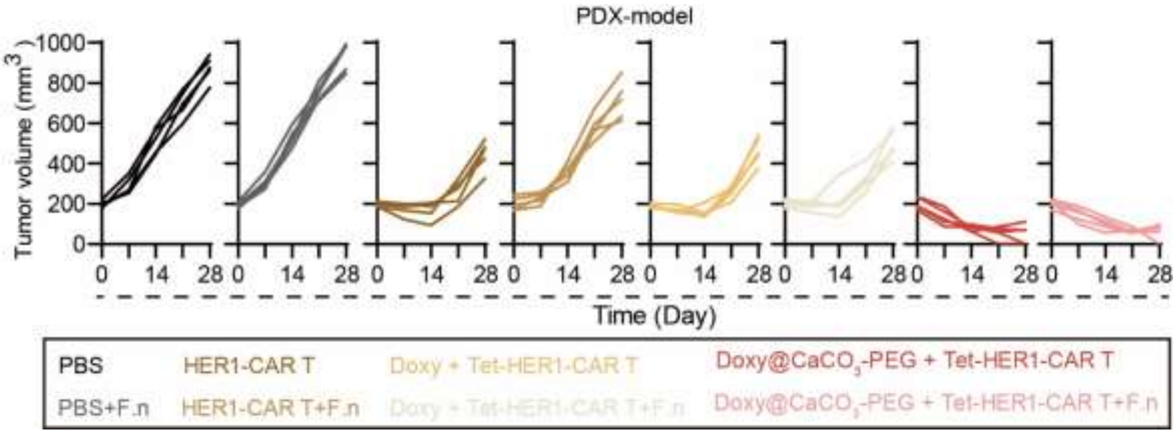

Figure S20. Individual tumor growth curves of TNBC PDX tumors in mice with different treatments as indicated.
